# *Fragaria viridis* Fruit Metabolites: Variation of LC-MS Profile and Antioxidant Potential during Ripening and Storage

**DOI:** 10.3390/ph13090262

**Published:** 2020-09-22

**Authors:** Daniil N. Olennikov, Aina G. Vasilieva, Nadezhda K. Chirikova

**Affiliations:** 1 Laboratory of Medical and Biological Research, Institute of General and Experimental Biology, Siberian Division, Russian Academy of Science, 6 Sakh’yanovoy Street, 670047 Ulan-Ude, Russia; 2 Department of Biology, Institute of Natural Sciences, North-Eastern Federal University, 58 Belinsky Street, 677027 Yakutsk, Russia; aina_vasilieva@mail.ru (A.G.V.); hofnung@mail.ru (N.K.C.)

**Keywords:** *Fragaria viridis*, creamy strawberry, ellagitannins, HPLC, mass spectrometry, fruit ripening, antioxidant potential

## Abstract

*Fragaria viridis* Weston or creamy strawberry is one of the less-known species of the *Fragaria* genus (Rosaceae family) with a wide distribution in Eurasia and is still in the shadow of more popular relatives *F. ananassa* (garden strawberry) or *F. vesca* (wild strawberry). Importantly, there is a lack of scientific knowledge on *F. viridis* compounds, their stability in the postharvest period, and bioactivity. In this study, metabolites of *F. viridis* fruits in three ripening stages were characterized with high-performance liquid chromatography with photodiode array and electrospray ionization triple quadrupole mass spectrometric detection (HPLC-PAD-ESI-tQ-MS). In total, 95 compounds of various groups including carbohydrates, organic acids, phenolics, and triterpenes, were identified for the first time. The quantitative content of the compounds varied differently during the ripening progress; some of them increased (anthocyanins, organic acids, and carbohydrates), while others demonstrated a decrease (ellagitannins, flavonols, etc.). The most abundant secondary metabolites of *F. viridis* fruits were ellagitannins (5.97–7.54 mg/g of fresh weight), with agrimoniin (1.41–2.63 mg/g) and lambertianin C (1.20–1.86 mg/g) as major components. Antioxidant properties estimated by in vitro assays (2,2-diphenyl-1-picrylhydrazyl radical (DPPH), 2,2′-azino-bis(3-ethylbenzothiazoline-6-sulfonic acid) cation radical (ABTS), ferric reducing antioxidant power (FRAP), and oxygen radical absorbance capacity (ORAC)) showed good antioxidant potential in all ripening stages of *F. viridis* fruits. The pilot human experiment on the effect of *F. viridis* fruit consumption on the serum total antioxidant capacity confirmed the effectiveness of this kind of strawberry. Postharvest storage of ripe fruits at 4 °C and 20 °C lead to declining content in the majority of compounds particularly ascorbic acid, ellagitannins, and flavonols, with the most significant loss at room temperature storage. These results suggest that *F. viridis* fruits are a prospective source of numerous metabolites that have potential health benefits.

## 1. Introduction

**Figure 1:**
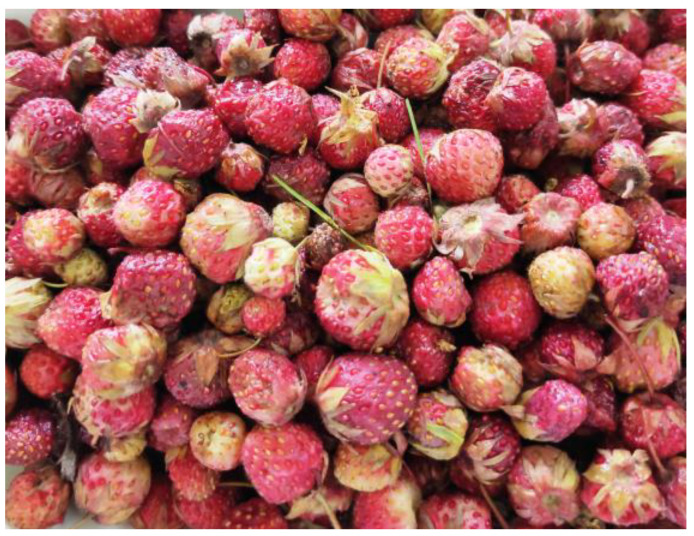
*Fragaria viridis* Weston (creamy strawberry).

Genus *Fragaria* (strawberry) of the Rosaceous family is a well-known source of dietary fruits that are popular all over the world and is widely consumed due to its unique taste and fragrance. The global production of strawberries has reached 9 million tons per year with maximal production levels in China, USA, and Mexico [1]. Such high consumption makes it necessary to examine the various aspects of biology, chemistry, cultivation, and technology of strawberry manufacturing. The most common studies devoted to the metabolic diversity of the *Fragaria* genus include many groups of compounds such as carbohydrates, organic acids, vitamins, anthocyanins, ellagitannins, flavonoids, and minerals [2]. Special attention is also paid to understanding the nature of postharvest changes in the chemical profile and physical properties of strawberry fruits in different storage conditions [3]. Despite the significance of strawberry as a food, pharmaceutical interest is also given due to the presence of various bioactive compounds including antioxidative [4], anti-inflammatory [5], antibacterial [6], anti-allergic [7], antidiabetic [8], and cancer preventive [9] properties. The antioxidative properties of various strawberries were discussed previously, and high effectiveness was revealed for *Fragaria* extracts and unprocessed fruits [10], thereby strengthening the interest to study strawberries. An age-old tradition of strawberry use resulted in the cultivation of many *Fragaria* species, not only popular species such as *F. ananassa* (garden strawberry), *F. vesca* (wild strawberry), and *F. moschata* (musk strawberry) but also exotic species like *F. chiloensis* (Chilean strawberry), *F. × bifera*, and *F. viridis* (creamy strawberry) [2]. In this regard, significant attention also needs to be focused on little-known strawberries, specifically *F. viridis* (Figure 1).

Botanically, *F. viridis* is a green perennial herbaceous rhizomatous plant that can grow up to 25 cm tall with numerous adventitious roots. The stems are erect, and the length of the leaves are slightly longer, densely dressed with protruding trichomes. The stipules are narrow and brown, and the leaves have shaggy petioles from prominent trichomes that densely cover them. The inflorescences are small, corymbose, loose, and few-flowered and are dressed at the base with a solid or tripartite apical leaf. The pedicels are short and dressed with appressed or occasionally horizontally protruding trichomes. The flowers are relatively large, up to 2.5 cm in diameter, usually bisexual with triangular and lanceolate sepals. The petals are rounded, overlapping each other, short-clawed, and yellowish-white. The fragrant fruits are spherical, narrowed at the base, mostly yellowish-white, only reddish at the top, and rarely entirely pink or pale red, with achenes slightly immersed in the pulp, which are difficult to separate from the receptacle [11]. The fruits are separated from the stem together with sepals at inconsist density, and they are distinguished by good transportability, better that *F. vesca* and *F. ananassa*. The species is ecologically plastic. It grows in aspen-birch groves, on open grassy mountain slopes, on edges and glades of mountain forests, in meadows, and in meadow steppes in Europe, Russia, the Caucasus, and Western and Eastern Siberia. Sensory evaluation of *F. viridis* fruits demonstrated good taste and extraordinary fresh-fruity flavour [12].

The literature data about *F. viridis* are meagre and demonstrated that the essential oil of leaves consists of major components β-linalool, *n*-nonanal, tetradecanal, nerolidol, α-bisabolol, and phytol, which distinguishes it from fruit essential oil with the dominant *m*/*p*-xylene, isoledene, methyleugenol, α-cedrene, α-muurolene, and α-cedrol [13]. The known phenolics of *F. viridis* fruits are catechin, epicatechin, epigallocatechin gallate, cyanidin 3-*O*-glucoside, pelargonidin 3-*O*-glucoside, quercetin 3-*O*-glucoside, ellagic acid [14], quercetin 3-*O*-galactoside, quercetin 3-*O*-rutinoside, and chlorogenic acid [15], equalling the phenolic profile of *F. viridis* leaves. Both fruits and leaf extracts showed good radical-scavenging and ferric-reducing ability [14]. Despite easy cultivation, high breeding potential, and good sensory parameters, *F. viridis* remains one of the underutilized strawberry species [12]. The growing interest in new strawberries as perspective food sources obliges us to do more in-depth research, particularly in the area of *Fragaria* metabolomics using high-performance liquid chromatography-mass spectrometric techniques (nothing has been done previously with these techniques). It is, of course, of great interest in revealing specificities of metabolite changes in *F. viridis* fruits during the ripening progress as well as metabolite transformation during fruit storage.

In the present report, we realized the first detailed metabolomic profiling of *F. viridis* fruits in three stages of ripening (unripe, intermediate ripe, and fully ripe) using high-performance liquid chromatography with photodiode array and electrospray ionization triple quadrupole mass spectrometric detection (HPLC-PAD-ESI-tQ-MS), and antioxidant properties of *F. viridis* fruits were also studied in four in vitro models (scavenging capacity against 2,2-diphenyl-1-picrylhydrazyl radical and 2,2′-azino-bis(3-ethylbenzothiazoline-6-sulfonic acid) cation radical, ferric reducing antioxidant power, and oxygen radical absorbance capacity) during ripening progress and one pilot human experiment (serum total antioxidant capacity). Finally, the variation of selected compounds and antioxidant potential of ripe *F. viridis* fruits was investigated in response to cool and room temperature storage. To our knowledge, this is the first comprehensive study of *F. viridis* fruits.

## 2. Results and Discussion

### 2.1. Metabolites of F. viridis Fruits: LS-MS Profile

**Figure 2:**
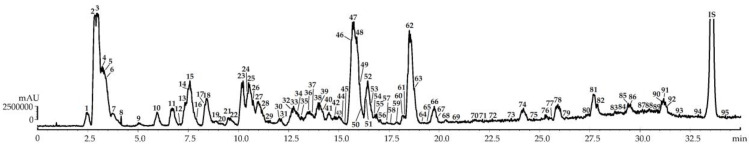
High-Performance Liquid Chromatography with Electrospray Ionization Triple Quadrupole Mass Spectrometric Detection (HPLC-ESI-tQ-MS) chromatogram (Total Ion Chromatogram (TIC) mode, negative ionization) of extract of *F. viridis* ripe fruits: compounds are numbered as listed in Table 1. IS—internal standard (genkwanin).

Chromatographic profiling of *F. viridis* fruit metabolites was completed by high-performance liquid chromatography with photodiode array and electrospray ionization mass spectrometric detection (HPLC-PAD-ESI-tQ-MS). The identification of components found in *F. viridis* was done after a precise interpretation of chromatographic (retention times) and spectral data (ultraviolet-visible spectra and mass spectral patterns) in comparison with reference standards and literature data. The extraction procedures of fresh fruits were preliminarily tested with various solvents (methanol, ethanol, isopropanol, water, and acetone), solvent–material ratios, temperatures (20–90 °C), and methods of extraction (ultrasound-, microwave-, and water-bath-assisted). The resultant protocol used was 100% methanol with a solvent–material ratio of 1:1 followed by 5 min homogenization and sonification (30 min, 45 °C). After comparison of *F. viridis* fruit extracts in three ripening stages (unripe, intermediate ripe, and fully ripe), the HPLC-ESI-tQ-MS chromatogram of fully ripe fruit extract showed the presence of the maximal amount of compounds (**95**) with interpretable data (Figure 2), details of which are provided in Table 1.

#### 2.1.1. Carbohydrates

Two types of carbohydrates were discovered in *F. viridis* fruits including hexosyl-hexose (*m*/*z* 341; **1**) and hexose (*m*/*z* 179; **2**). The HPLC-MS method used does not allow for identification of the nature of carbohydrates, so we used the HPLC-DAD procedure demonstrating the presence of three compounds identified as glucose, fructose, and saccharose (Figure S1), usual mono- and disaccharides of strawberry fruits [2].

#### 2.1.2. Organic Acids

Citric (**3**), malic (**4**), tartaric (**5**), fumaric (**6**), ascorbic (**7**), and oxalic acids (**8**) were the organic acids found in *F. viridis* fruits. All mentioned compounds were shown previously in *F. ananassa* [33] and *F. vesca* [34].

#### 2.1.3. Gallic Acid Derivatives

Gallic acid (**10**), 1-*O*-glucoside (**9**), and 1,2,3,4,6-penta-*O*-galloylglucose (**67**) were identified by comparison with reference standards. Gallic acid derivatives and gallotannins are not typical phenolics of *Fragaria* genus, but gallic acid is a metabolite found in *F. ananassa* [2] and compound **67** is detected in *F. ananassa* fruits [35] and *F. vesca* leaves [36].

#### 2.1.4. Ellagic Acid Derivatives and Ellagitannins

Ellagic acid (**53**), four ellagic acid glycosides (**41**, **43**, **82**, and **86**), and eighteen ellagitannins (**11**, **14**, **16**, **18**, **30**, **33**–**35**, **40**, **42**, **44**, **46**, **47**, **51**, **52**, **54**, **62**, and **63**) were found in *F. viridis* fruits. The ellagic acid glycosides were ellagic acid *O*-pentoside (**41**), ellagic acid *O*-desoxyhexoside (**43**), ellagic acid *O*-methyl ester-*O*-desoxyhexoside (**82**), and ellagic acid di-*O*-methyl ester-*O*-desoxyhexoside (**86**) due to the presence of ions with *m*/*z* 301 typical for ellagic acid derivatives [18,23] and the size of loss particles with *m*/*z* 132 (*O*-pentose) or 146 (*O*-desoxyhexose) [16].

Known strawberry ellagitannins, lambertianin C (**47**), sanguiin H10 (**42**), sanguiin H6 (**52**; **46** as isomer), sanguiin H2 (**35**; **44** as isomer), and pedunculagin (**11**; **18** as isomer), were identified using reference standards [18]. The literature data gave additional identification of three *Fragaria* ellagitannin structures: strictinin (**14** and **16**), castalagin (**30** and **34**), and casuarictin (**33** and **40**) [18]. Agrimoniin (**62**) gave typical ions of deprotonated fragment [M-H]^−^ (*m*/*z* 1869) and double-charged particle [M-2H]^2^^−^ (*m*/*z* 934). The further MS/MS fragmentation of molecule **62** demonstrated the loss of fragments of hexahydroxydiphenoyl (HHDP; 302 Da), *bis*-HHDP-glucose (*bis*-HHDP-Glc; 784 Da), and galloyl-*bis*-HHDP-glucose (Gall-*bis*-HHDP-Glc; 934 Da), creating a cascade of ions with *m*/*z* 1567 [(M-H)-HHDP]^−^, 1265 [(M-H)-2×HHDP]^−^, 1085 [(M-H)-(*bis*-HHDP-Glc)]^−^, 935 [(M-H)-(Gall-*bis*-HHDP-Glc)]^−^, 633 [(M-H)-(Gall-*bis*-HHDP-Glc)-HHDP]^−^, and 481 [((*bis*-HHDP-Glc)-H)-HHDP]^−^ [23,29]. Agrimoniin was previously identified as the main ellagitannin of strawberry fruits from *F. vesca* and *F. ananassa* [24] and were found in *F. viridis* for the first time in this study. Compound **63** with a [M-2H]^2^^−^ ion with *m*/*z* 1018 gave the weak deprotonated ion [M-H]^−^ (*m*/*z* 2037); after decarboxylation (−44 Da) and loss of HHDP, gave the fragment with *m*/*z* 1691; and then degraded to fragments with *m*/*z* 1567 (loss of trihydroxy benzene) and 1265 (loss of HHDP). The alternative pathway of fragmentation of the particle with *m*/*z* 1691 led to the formation of de-HHDP-glucosylated ion with *m*/*z* 1209 and the fragments with *m*/*z* 935 [Gall-*bis*-HHDP-Glc–H]^−^, 783 [(*bis*-HHDP-Glc)-H]^−^, 633 [((Gall-*bis*-HHDP-Glc)-H)-HHDP]^−^, and 481 [((*bis*-HHDP-Glc)-H)-HHDP]^−^. The close mass spectrometric pattern gave the known strawberry ellagitannin fragariin A found in *F. ananassa* and has the structure of galloylated derivative of agrimoniin [29]. Two isomeric ellagitannins, **51** and **54**, were identified as biogenetic relatives to agrimoniin compounds, agrimonic acids A and B, respectively [28].

After all, only ellagic acid was mentioned previously as a component of *F. viridis* [14], indicating that this is the first report describing the profile of ellagic acid derivatives from *F. viridis* fruits. Traditionally used strawberries, such as *F. ananassa* and *F. vesca*, are a good source of ellagitannins and ellagic acid glycosides [2,18]. Lambertianin C, saguiins, and agrimoniin were also found in many varieties of cultivated strawberries, demonstrating their obligate position in the *Fragaria* metabolome [2,18,26,27].

#### 2.1.5. Hydroxycinnamates and Coumarins

Four known hydroxycinnamates were identified by comparison with reference standards, including 4-*O*-caffeoylquinic acid (**13**), 5-*O*-caffeoylquinic acid (**15**), 3-*O*-caffeoylquinic acid (**17**), and *p*-coumaric acid 4-*O*-glucoside (**25**). Only 5-*O*-caffeoylquinic acid was previously found in *F. viridis* [15]. Component **24** produced a deprotonated ion with *m*/*z* 325 and dehexosylated fragment with *m*/*z* 163 characteristic for *p*-coumaric acid *O*-hexoside [22]. One coumarin umbelliferone (**12**) was also identified by comparison with a reference standard.

#### 2.1.6. Catechins and Procyanidins

Catechin (**23**) and procyanidins B2 (**19**), B4 (**21**), and C2 (**31**) as well as an isomer to **31** catechin/epicatechin trimer **36** were detected in *F. viridis* fruits. Monomer **23** was already found in whole plant *F. viridis* [14].

#### 2.1.7. Anthocyanins

Derivatives of cyanidin (**20**, **26**, **27**, **32**, and **38**) and pelargonidin (**22**, **28**, **29**, **37**, and **48**) were found in *F. viridis* fruits based on UV-Vis patterns (525–535 nm for cyanidins and 498–505 nm for pelargonidins) and mass spectral behaviour of aglycone fragments (*m*/*z* 285 for cyanidins and 269 for pelargonidins). Five phenolics were identified after comparing spectra with reference standards: cyanidin-3-*O*-sophoroside (**20**), cyanidin-3-*O*-rutinoside (**26**), cyanidin-3-*O*-glucoside (**27**), pelargonidin-3-*O*-rutinoside (**28**), and pelargonidin-3-*O*-glucoside (**29**). Anthocyanin *O*-glucosides **27** and **29** are the most frequent phenolic pigments of *Fragaria* fruits [2,14,26], and *O*-rutinoside **28** was found in *F. ananassa* [26]. Compound **22** gave an [M-2H]^−^ ion with *m*/*z* 593 and two dehexosylated fragments with *m*/*z* 431 [(M-2H)-hexose]^−^ and 269 [(M-2H)-2×hexose]^−^ and was determined as pelargonidin di-*O*-hexoside. The closest phenolic to **22** is pelargonidin-3-*O*-sophoroside detected in *F. ananassa* [27]. Two compounds, **32** and **37,** have additional maxima in UV spectra at approximately 312 nm characteristic to acylic anthocyanins with *p*-coumaroyl fragments [37], and they were preliminary determined as cyanidin *O*-*p*-coumaroyl-*O*-hexoside (**32**) and pelargonidin *O*-*p*-coumaroyl-*O*-hexoside (**37**). Two mono-acetylated *O*-hexosides, **38** and **48,** gave mass spectral ion fragments with *m*/*z* 42 and 162 and were identified as cyanidin *O*-acetyl-*O*-hexoside (**38**) and pelargonidin *O*-acetyl-*O*-hexoside (**48**). The known acylated anthocyanins of *Fragaria* species traditionally have moieties of acetic and malonic acids [2,26,27,34] so the *p*-coumaroyl esters of anthocyanins were found in *Fragaria* fruits for the first time.

#### 2.1.8. Flavonols

Flavonols were defined by their specific UV spectral patterns with absorption at 256/268/360 nm for quercetin derivatives and 265/343 nm for kaempferol derivatives [17]. Thirty-four compounds were flavonols found in *F. viridis* fruits including two aglycones—quercetin (**74**) and kaempferol (**76**)—and 32 glycosides with non-acylated and acylated fragments linked with carbohydrate moieties.

Quercetin glycosides were the most diverse group of *F. viridis* phenolics with 19 members, some of which were identified using standard references. There are non-acylated compounds, such as quercetin-3-*O*-sophoroside (**39**), quercetin-3-*O*-rutinoside (**45**; rutin), quercetin-3-*O*-glucoside (**49**; isoquercitrin), quercetin-3-*O*-glucuronide (**50**; miquelianin), quercetin-3-*O*-xyloside (**55**; reynoutrin), and quercetin-3-*O*-arabinoside (**56**; avicularin) as well as acylated derivatives quercetin-3-*O*-(6″-*O*-*p*-coumaroyl)-glucoside (**60**; helichrysoside), quercetin-3-*O*-(6″-*O*-malonyl)-glucoside (**65**), quercetin-3-*O*-(2″-*O*-acetyl)-glucoside (**70**), quercetin-3-*O*-(6″-*O*-acetyl)-glucoside (**71**), and quercetin-3-*O*-(2″,6″-di-*O*-acetyl)-glucoside (**79**). Compounds **45**, **49**, and **74** were reported in *F. viridis* by Raudonis et al. [14] and in *F. ananassa* by many authors [26,27,35]. The remaining quercetin glycosides were acylated derivatives of quercetin *O*-hexoside giving the same MS/MS fragments with *m*/*z* 463 (quercetin *O*-hexoside) and 301 (aglycone). Compound **61** had [M-H]^−^ at *m*/*z* 609 and MS/MS fragmentation close to **60**, making it an isomer with the most likely structure of quercetin *O*-*p*-coumaroyl-*O*-hexoside. Glycoside **64** gave deprotonated ions with *m*/*z* 549 and is an isomer of quercetin 3-*O*-(6″-*O*-malonyl)-glucoside (**65**) or quercetin *O*-malonyl-*O*-hexoside.

A series of mixed *O*-acylated quercetin *O*-hexosides had higher retention times than quercetin. Their specific MS patterns showed the loss of fragments with *m*/*z* 42 (acetyl), 86 (malonyl), and/or 146 (*p*-coumaroyl). Five combinations of acylated quercetin *O*-hexosides were found, such as acetyl/malonyl (**80**), malonyl/*p*-coumaroyl (**84** and **85**), acetyl/*p*-coumaroyl (**88**), di-acetyl/*p*-coumaroyl (**94**), and acetyl/malonyl/*p*-coumaroyl (**95**). To date, there are no known analogues of compounds **80**, **84**, **85**, **88**, **94,** and **95**, but most likely, structures are quercetin-3-*O*-glucosides with a substituted glucose moiety at positions 2″, 3″, 4″, and 6″.

Thirteen kaempferol glycosides were found in *F. viridis* fruits, and four were partially identified using a comparison of *t*_R_, UV, and mass spectrometric data with reference standards. There were kaempferol-3-*O*-rutinoside (**57**; nicotiflorin), kaempferol-3-*O*-glucoside (**58**; astragalin), kaempferol-3-*O*-glucuronide (**59**), kaempferol-3-*O*-(6″-*O*-*p*-coumaroyl)-glucoside (**66**; tiliroside), and kaempferol-3-*O*-(6″-*O*-malonyl)-glucoside (**69**). No kaempferol glycosides were previously found in *F. viridis*, but other *Fragaria* species were reported to contain compounds **57**, **58**, **59**, and **66** (*F. ananassa*, *F. vesca*, and *F. chiloensis*) [2]. Non-mixed acylated kaempferol *O*-hexosides were defined as kaempferol *O*-malonyl-*O*-hexosides (**68**), kaempferol *O*-acetyl-*O*-hexosides (**72**,**73**), and kaempferol di-*O*-acetyl-*O*-hexoside (**87**). Among the possible known analogs of observed flavonols, kaempferol-3-*O*-(2″-*O*-malonyl)-glucoside (for **68**), kaempferol-3-*O*-(6″-*O*-acetyl)-glucoside (for **72**,**73**), and kaempferol-3-*O*-(3″,4″-di-*O*-acetyl)-glucoside (for **87**) should be mentioned [38]. Mixed acylated kaempferol *O*-hexosides were also found in *F. viridis* extract and identified using the same principle as quercetin *O*-hexosides; these compounds include kaempferol *O*-acetyl-*O*-malonyl-*O*-hexoside (**89**), kaempferol *O*-malonyl-*O*-*p*-coumaroyl-*O*-hexosides (**90** and **92**), and kaempferol *O*-acetyl-*O*-*p*-coumaroyl-*O*-hexoside (**93**). Contrary to mixed quercetin *O*-hexosides, there are some known variants of mixed kaempferol *O*-hexosides like kaempferol-*O*-3-(3″/4″-*O*-acetyl-6″-*O*-*p*-coumaroyl)-glucosides as an alternative for **93** [38].

#### 2.1.9. Triterpenes

Six compounds were triterpenes, and two reference standard defined compounds were tormentic acid (**81**) and pomolic acid (**91**). Both compounds are the usual Rosaceous metabolites [39,40] but were not found in *Fragaria* fruits early studies. Glycosidic derivatives of **81** and **91** were described as two *O*-hexosides, **78** and **83,** and two di-*O*-hexosides, **75** and **77,** and are unusual components of strawberry fruits.

### 2.2. Quantitative Content and LS-MS Profile Variation of F. viridis Fruits during Ripening

We studied *F. viridis* fruits at three different stages of ripening, including unripe fruits, the stage of technological ripeness (intermediate stage), and the stage of full ripeness (Table 2). The total simple carbohydrate (mono- and disaccharides) content in *F. viridis* fruits varied from 41.14 mg/g in the unripe stage to 45.17 mg/g in ripe fruits. The main components were monosaccharides, glucose, and fructose, with a concentration of 41.10–45.16 mg/g responsible for the sweet taste of *F. viridis* fruits. Monosaccharides are the dominant sugars of *F. ananassa* [33,41,42] and *F. vesca* [43], but in some strawberry varieties, it happens that saccharose shows the highest content [34,44]. The sugar content of *F. ananassa* fruits demonstrated the same trend during ripening, with the lowest content in unripe fruits (3.61–4.45 mg/g) rising to the ripe stage with 4.82–8.20 mg/g [33], indicating the close character of carbohydrates changing in strawberries.

The highest total content of organic acids was found in ripe fruits of *F. viridis* (7.88 mg/g) and the lowest was found in the unripe stage (4.25 mg/g) including the greatest share of citric acid in all stages of ripening (2.83–5.63 mg/g). The remaining organic acids were minor components with concentration values 0.42–0.59 mg/g for malic acid, 0.37–0.42 mg/g for tartaric acid, 0.01–0.07 mg/g for fumaric acid, and trace–0.05 mg/g for oxalic acid. This results in an increase in the acidity of *F. viridis* fruits during ripening, which had a positive impact on strawberry taste. The domination of citric acid was demonstrated previously in many *F. ananassa* cultivars grown in Slovenia (4.4–10.5 mg/g) [42], Pakistan (12.0–14.3 mg/g) [33], and Turkey (5–10 mg/g) [45] as well as in *F. vesca* (5.6 mg/g) [34]. Moreover, the fruit development resulted in an increase in organic acids in strawberries [33], and malic, tartaric, fumaric, and oxalic acids were the minor acids in other *Fragaria* fruits [33,34,45]. Particularly noteworthy was the presence of a high level of ascorbic acid, up to 1.12 mg/g in ripe *F. viridis* fruits, that was significantly more than found in *F. vesca* (0.4 mg/g) and *F. ananassa* (0.25–0.9 mg/g) [33,45].

Galic acid derivatives showed trace (gallic acid, 1,2,3,4,6-penta-*O*-galloylglucose) or low-level content (1-*O*-galloyl glucose) without significant variation during ripening (0.04–0.05 mg/g). In contrast to the gallic acid derivatives, hydroxycinnamates were important compounds of *F. viridis* fruits with medium content although the amount decreased during ripening from 0.97 mg/g to 0.60 mg/g. The highest level was found for the *p*-coumaric acid 4-*O*-glucoside (0.29–0.35 mg/g), its isomer **24** (0.08–0.14 mg/g), and 5-*O*-caffeoylquinic acid (0.14–0.28 mg/g). The early study of 5-*O*-caffeoylquinic acid content variation in dry strawberries showed it ranging from 1.8 to 2.9 mg/g for *F. vesca*, from 1.2 to 1.7 mg/g for *F. viridis*, and from 0.7 to 1.8 mg/g for *F. moschata* [15]. The level of coumaroyl glycosides in Norway *F. ananassa* cultivars varied from 0.02 mg/g to 0.14 mg/g with maximal content in fully ripe fruits [25], which is different from our findings.

The most significant groups of phenolics with the highest content in all stages of maturity of *F. viridis* fruits were ellagitannins and ellagic acid derivatives. The general rule of ellagitannin variation in *F. viridis* fruits (with few exceptions) was decreasing content during ripening. This was particularly manifested in dominant compounds of agrimoniin (2.63→1.41 mg/g), lambertianin C (1.86→1.20 mg/g), fragariin A (0.93→0.63 mg/g), and sanguiin H6 (0.36→0.22 mg/g), where values were maximal in unripe fruits. The decrease of ellagitannin level in strawberry fruits during development was previously shown in some Norway cultivars of *F. ananassa*; the variation of agrimoniin content in Blink, Polka, and Senga cultivars was 0.72→0.57, 0.66→0.58, and 0.68→0.55 mg/g, respectively [25]. The most drastic fall in ellagitannin content, from 1.14 to 0.30 mg/g, was found in Italian cultivars of *F. ananassa* [18]. The most likely reason for the ellagitannin changes is due to increasing activity of specific enzymes, such as tannases, reaching the highest values in ripening fruits [46].

This is further illustrated by the slight rising content of ellagic acid, some ellagic acid *O*-glycosides, and low molecular weight ellagitannins (as pedunculagin, strictinin, castalagin, and casuarictin) that can be considered the breakdown products of ellagitannin molecules, but of course, this issue needs to be discussed additionally.

Catechins and procyanidins are compounds with medium levels in *F. viridis* fruits, for which the total concentration decreased from unripe to the ripe stage (0.29→0.09 mg/g). The content of major components showed the same behaviour, including catechin (0.11→0.05 mg/g), procyanidin B2 (0.09→0.02 mg/g), and procyanidin C2 (0.05→0.01 mg/g). Aaby et al. [25] reported similar data for 27 cultivars of *F. ananassa* for the content of catechin (0.02–0.08 mg/g), procyanidin dimers (0.05–0.16 mg/g), and trimers (0.05–0.19 mg/g).

Anthocyanins, which are important phenolics of strawberries, were at a low level in *F. viridis* characterized by slight red pigmentation of the outer layer of fruits and depigmented pulp. It is for this reason that the unripe and pre-ripe fruits had trace anthocyanin content. In the ripe stage, the domination of pelargonidin 3-*O*-glucoside (0.06 mg/g), cyanidin 3-*O*-glucoside (0.05 mg/g), and pelargonidin 3-*O*-rutinoside (0.03 mg/g) was observed. The remaining pigments were found in trace levels. Pelargonidin and cyanidin glycosides were also found as components of phenolic pigments of all studied strawberries including *F. ananassa* [47], *F. vesca,* and *F. moschata* [14]. It is to be expected that the ripening of strawberry fruits resulted in intense pigmentation caused by the accumulation of anthocyanins, as in the case of Norway cultivars from 0.2 mg/g in the pre-ripe stage to 0.8 mg/g in fully ripe fruits of *F. ananassa* [25].

The total concentration of flavonols in *F. viridis* demonstrated decreasing levels during fruit development from 1.24 mg/g in unripened fruits to 1.01 mg/g in ripened fruits. Quercetin derivatives (0.66–0.82 mg/g) prevailed over kaempferol derivatives (0.35–0.42 mg/g) in all stages of ripening; this was also found in *F. ananassa* (0.01–0.05 mg/g for quercetin derivatives vs. 0.01–0.02 mg/g for kaempferol derivatives) [9]. The main components of flavonol complex of *F. viridis* were quercetin 3-*O*-rutinoside (0.25–0.32 mg/g) and kaempferol 3-*O*-rutinoside (0.11–0.28 mg/g), followed by two acylated compounds: quercetin 3-*O*-(6″-*O*-*p*-coumaroyl)-glucoside (0.05–0.11 mg/g) and kaempferol 3-*O*-(6″-*O*-*p*-coumaroyl)-glucoside (0.04–0.08 mg/g). The gradual decline of flavonoid concentration was found for flavonol di-*O*-glycosides and acylated flavonol *O*-glycosides in contrast to aglycones and flavonol mono-*O*-glycosides accumulated in ripe fruits. Again, the variation of enzymatic activity of hydrolases may be relevant in the progress of *F. viridis* fruits ripening. This phenomenon has not been mentioned previously in any strawberries and needs additional experimental data to confirm this finding.

Triterpenes found in *F. viridis* fruits were trace compounds, but despite this, the quantifiable levels of tormentic acid, its *O*-hexoside, and pomolic acid were found in the stage of full ripening. The variation of triterpenoids in any *Fragaria* species was not discussed previously, but it is known that the accumulation of triterpenoids reaches the maximal level in the fully ripe stage of fruits; this was also declared for Chardonnay grape [48], olive fruits [49], and tomato [50].

### 2.3. Antioxidant Potential of F. viridis Fruits: Comparision with Other Strawberries

The activity of fruit total extracts of *F. viridis* in three stages of ripening was studied in four antioxidant assays including the scavenging capacity against 2,2-diphenyl-1-picrylhydrazyl radical (DPPH), and 2,2′-azino-bis(3-ethylbenzothiazoline-6-sulfonic acid) cation radical (ABTS), ferric reducing antioxidant power (FRAP), and oxygen radical absorbance capacity (ORAC) (Table 3). In comparison, the activity of two extracts from commercially available ripe fruits of *F. vesca* (wild strawberry, *Regina* cultivar) and *F. ananassa* (garden strawberry, *Senga Sengana* cultivar) was also studied. Both species are much more common strawberries, and their antioxidant potential has been analysed many times [2].

The extracts of *F. viridis* fruits in all stages of ripening were effective radical scavengers against both radicals DPPH and ABTS. Variations of the antioxidant potential values were 27.53–29.18 μM trolox-eq./g in the DPPH assay and 35.07–36.22 μM trolox-eq./g in the ABTS assay, while the more active scavenger in the DPPH assay was the extract of unripe fruits and the ABTS assay gave the extract of ripe fruits as more active. The extracts of *F. vesca* and *F. ananassa* were less effective in DPPH/ABTS scavenging assays with values of trolox-equivalent content 15.21/19.73 and 9.33/14.67 μM/g, respectively. An early research of *F. ananassa* extracts in the DPPH assay showed a wide range of fluctuation of antiradical activity from 9.75–12.83 μM BHT-eq./g for Brazil cultivars [4] to 3.00–13.15 μM trolox-eq./g for Polish cultivars [10]. Sikmilar characteristics were found for ABTS assay data varying from 1.50–2.27 μM trolox-eq./g for Japan varieties [51] to 7.06–29.73 μM trolox-eq./g for Polish cultivars [10]. Raudonis et al. [14] analysed the activity of *F. vesca* and *F. moschata* extracts using HPLC-assisted ABTS assay, which gave the values of protection 25.11 and 8.24 μM trolox-eq./g, respectively.

The FRAP assay is one of the most popular assays to estimate antioxidant activity for analysis of edible fruits, and strawberries are not an exception. The level of ferric reducing antioxidant power of *F. viridis* extracts was high and increased during fruit ripening from 42.63 μM trolox-eq./g in the unripe stage to 47.11 μM trolox-eq./g in ripe fruit extract. The parameters of *F. vesca* (27.14 μM trolox-eq./g) and *F. ananassa* (21.06 μM trolox-eq./g) extracts were lower but close to known data (24.84 μM trolox-eq./g for *F. vesca* [14]). The level of antioxidant activity in the ORAC assay for *F. viridis* fruit extracts was similar to ABTS data and slightly decreased in ripening progress from 33.62 μM trolox-eq./g (unripe fruits) to 32.98 μM trolox-eq./g (ripe fruits). The ORAC data of *F. vesca* extract 25.05 μM trolox-eq./g was higher than for *F. ananassa* extract (18.87 μM trolox-eq./g) but at a level lower than *F. viridis*. The known information about ORAC potential of strawberries demonstrated high effectiveness of anthocyanins fraction of *F. ananassa* (2.7–24.46 mM trolox-eq./g) [52], opposite the activity of the total fruit extract (8.90–16.63 μM trolox-eq./g) [53].

Applying the DPPH-radical scavenging-assisted HPLC-PDA-ESI-tQ-MS assay, we identified the compounds responsible for the antioxidant defence of *F. viridis* extracts. For that to happen, an aliquot of the total extract was separated with chromatography and portions of the eluates were collected. A part of the eluates was used for the DPPH decolouration assay, and the remainder were used for HPLC-PDA-ESI-tQ-MS assay for the qualitative confirmation of compounds (Figure S2). The results showed that the majority of compounds found in *F. viridis* were involved in the process of radical scavenging (most likely because of their phenolic nature), but 12 sites of elution gave more pronounced decolouration of the DPPH solution. There were ascorbic acid, ellagic acid, five ellagitannins (pedunculagin, sanguiin H6, lambertianin C, agrimoniin, and fragariin A), two anthocyanins (pelargonidin 3-*O*-glucoside, and cyanidin 3-*O*-glucoside), and three flavonols (quercetin 3-*O*-glucoside, quercetin 3-*O*-glucuronide, and quercetin 3-*O*-rutinoside). Three compounds, ascorbic acid, lambertianin C, and agrimoniin, were the most active due to their high content so they were the principal antioxidants of *F. viridis* fruits.

**Figure 3:**
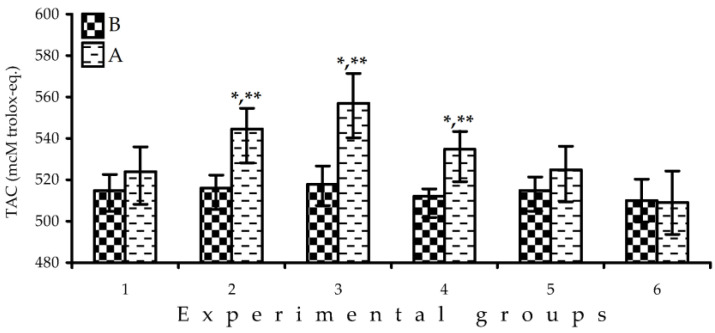
Changes in serum total antioxidant capacity (TAC) before (B) and after (A) 1-week intake of *Fragaria* fresh ripe fruits (group 1—*F. viridis*, 100 g/day, *n* = 5; group 2—*F. viridis*, 250 g/day, *n* = 5; group 3—*F. viridis*, 400 g/day, *n* = 5; group 4—*F. vesca*, 250 g/day, *n* = 4; and group 5—*F. ananassa*, 250 g/day, *n* = 6) and 10 g/day fructose (group 6, control group; *n* = 3). * *p* < 0.05 vs. control group after intake; ** *p* < 0.05 vs. same group before intake.

In brief, the information obtained in four in vitro assays demonstrates the high effectiveness of *F. vesca* fruit extracts as antioxidant agents, exceeding the activity of two well-known strawberries: *F. vesca* and *F. ananassa*. In light of the obtained results about the activity of *F. vesca* fruits, checking its usefulness in antioxidant protection of the human organisms was considered. To this end, this was done by pilot experiment by analysing total antioxidant capacity (TAC) of blood serum of healthy male volunteers after a 1-week intake of *F. viridis* fresh ripe fruits at doses of 100, 250, and 400 g/day. The level of TAC before *F. vesca* fruit intake was 510–516 μM trolox-eq./L. The week-long consumption of *F. viridis* fresh fruits gave a statistically significant increase of serum TAC level in all dose groups up to 524, 544, and 557 μM trolox-eq./L, respectively, for groups with 100, 250, and 400 g/day consumption (Figure 3).

By comparison, the results of *F. vesca* and *F. ananassa* fruit groups (both 250 g/day) were also positive—the consumption of both kinds of strawberries resulted in increases in serum TAC levels, but to a lesser degree (535 μM trolox-eq./L for *F. vesca*, 525 μM trolox-eq./L for *F. ananassa*).

This illustrates the good antioxidant potential of *F. viridis* fruits in any dose applied. To date, it is known that the consumption of *F. ananassa* resulted in increases in serum TAC by 7–25% [54]. The possible reasons for that phenomenon may be an increase in human serum of strawberry-related metabolites such as pelargonidin-glucuronide, urolithin A-glucuronide [55] and *p*-hydroxybenzoic acid [56] possessing high antioxidant potential and also the rising of serum antioxidants (glutathione) and serum level of antioxidant enzyme activity (catalase, glutathione peroxidase, and glutathione reductase) [57]. The metabolite profile of *F. viridis* is qualitatively and quantitatively close to *F. ananassa*; therefore, it is logical to assume that the increase of serum TAC after *F. viridis* consumption is caused by enhancement of the serum level of antioxidant metabolites of phenolic nature, serum antioxidants, and antioxidant enzymes.

### 2.4. Storage Stability of Antioxidants and Antioxidant Potential of F. viridis Ripe Fruits

Twenty compounds with the most pronounced antioxidant effects were quantified in *F. viridis* fruits in two series of storage experiments (Table 4). Primarily, we studied the change in concentration of ripe fruits stored at two temperatures, 4 °C (cool temperature) and 20 °C (room temperature), to define the stability of antioxidants in fresh fruits for a short period.

The seven-day storage of ripe *F. viridis* fruits at 4 °C and three-day storage at 20 °C were the maximal periods of storage without external damage (browning, rotting, untypical smell, and taste) [58].

Results from HPLC data show that the storage of ripe *F. viridis* fruits at 4 °C caused a decrease of ascorbic acid content by 55.2% (1.14→0.51 mg/g), anthocyanin content by 28.6–60.0% (0.07→0.05 mg/g for pelargonidin 3-*O*-glucoside and 0.05→0.02 mg/g for cyanidin-3-*O*-glucoside), ellagitannin polymers content by 20.4–26.2% (1.26→0.93 mg/g for lambertianin C, 1.47→1.17 mg/g for agrimoniin, and 0.65→0.51 mg/g for fragariin A), and quercetin 3-*O*-rutinoside content by 16.7% (0.24→0.20 mg/g). There has also been an increase in the concentration of ellagic acid by 90% (0.10→0.19 mg/g), ellagitannin monomers and dimers by 17.5–25.0% (0.33→0.40 mg/g for pedunculagin and 0.20→0.25 mg/g for sanguiin H6), and flavonol monoglucosides by 10.0–13.3% (0.10→0.11 mg/g for quercetin 3-*O*-glucoside and 0.15→0.17 mg/g for quercetin 3-*O*-glucuronide). As a result of chemical changes, the reduction of bioactivity of fruits was also observed, and the loss of total antioxidant potential was 20.6% (4.12→3.27 μmol trolox-eq./g).

The storage of fresh *F. viridis* fruits at room temperature (20 °C) resulted in more drastic changes within a shorter period. The level of ascorbic acid declined from 1.14 mg/g to 0.36 mg/g (68.4%) for three days; additionally, anthocyanins became a trace compound. The strong reduction of content was detected for the polymeric ellagitannins, lambertianin C (42.6%), agrimoniin (36.7%), and fragariin A (66.2%) in opposition to ellagic acid, pedunculagin, and sanguiin H6, which increased at 150.0, 48.5, and 50.0%, respectively. The flavonoid biocide quercetin 3-*O*-rutinoside showed a statistically significant decrease of content from 0.24 mg/g to 0.18 mg/g (25%) coupled with a rising level of quercetin 3-*O*-glucoside and quercetin 3-*O*-glucuronide.

The parameter of total antioxidant potential decreased from 4.12 to 0.52 μmol trolox-eq./g or 87.4% less antioxidant potential. Postharvest storage of ripe fruits is inextricably linked to senescence causing changes in biochemical profiles, biomolecules and polymers degradation, cell dysfunction and disintegration, and the leaking of enzymes [58]. Not long after, the fruits begin rotting, which reduces its alimentary value. In our study, the ripe *F. viridis* fruits after storage showed negative changes in content of ascorbic acid and anthocyanins, which are environmentally unstable plant compounds diminished in light and high humidity [59,60], just like polymeric ellagitannins and rutin typically degrading after contact with oxygen and esterase-like enzymes [61,62]. The preservative value of cool temperature (4 °C) was higher than room temperature (20 °C), saving antioxidants and the antioxidant potential of fruits longer. The decrease in phenolic compounds and ascorbic acid content in strawberries was shown in the number of papers. Anthocyanin content decreased in *F. ananassa* fruits during refrigerated storage at 4 °C in cultivars Camarosa (385→46 mg/kg) [63,64] and Elsanta (40→20 mg/g) [65]. The ascorbic acid level was also unstable at 0–20 °C with a loss of about 40% (cultivars Dover, Campineiro, and Mazi) [3] or more (cultivar Camarosa) [63]. The content of ellagic acid and flavonol monoglucosides in cool storage (5 °C) of *F. ananassa* fruits tends to rise as in the Selva cultivar from 19.9 to 26.8 μg/g for ellagic acid, from 40.1 to 44.1 μg/g for quercetin derivatives, and from 13.7 to 15.8 μg/g for kaempferol derivatives [66]. Our findings revealed that various strawberries (*F. viridis* and *F. ananassa*) have the same response during storage at cool and room temperature conditions.

## 3. Materials and Methods

### 3.1. Plant Materials and Chemicals

Samples of *Fragaria viridis* fruits were collected in Sakha (Yakutia) Republic (Aldanskii ulus, 58°37′27.1″ N, 125°17′17.5″ E, 15–25 July 2019) in three ripening stages (unripe—green fruits, intermediate ripe—half red fruits, and fully ripe—red fruits). The species was authenticated by N.I. Kashchenko (IGEB SB RAS, Ulan-Ude, Russia). The fruits were conditioned in plastic boxes and transported to the laboratory at 4 °C within 2–3 h. The ripe fruits of *F. vesca* (*Regina* cultivar) and *F. ananassa* (*Senga Sengana* cultivar) were purchased via a local market. The reference compounds were purchased from BioCrick (Chengdu, PRC), BOC Sciences (Shirley, NY, USA), Carbosynth Ltd. (Compton, UK), ChemFaces (Wuhan, PRC), Extrasynthese (Lyon, France), Funakoshi Co. Ltd. (Tokyo, Japan), Sigma-Aldrich (St. Louis, MO, USA), Toronto Research Chemicals (North York, ON, Canada), and TransMIT GmbH (Gießen, Germany) (Table S1). Ellagitannins sanguiins H2, H6, and H10; agrimonic acids A and B; and agrimoniin were isolated previously in our laboratory from Rosaceous species (purity 90–95%) [28,67,68], and flavonols quercetin 3-*O*-(2″-*O*-acetyl)-glucoside, quercetin 3-*O*-(2″-*O*-acetyl)-glucoside, and quercetin 3-*O*-(2″,6″-di-*O*-acetyl)-glucoside were isolated from *Calendula officinalis* [32]. Selected chemicals were from Sigma-Aldrich—acetonitrile for HPLC (Cat. No 34851, ≥99.9%), 2,2′-azino-bis(3-ethylbenzothiazoline-6-sulfonic acid) diammonium salt (Cat. No A1888, ≥98%), 2,2′azobis(2-methylpropionamidine) dihydrochloride (Cat. No 440914, ≥97%), 2,2-diphenyl-1-picrylhydrazyl radical (Cat. No 281689, ≥97%), formic acid (Cat. No 33015, ≥98%), fructose (Cat. No 47739, ≥99%), hydrogen peroxide (Cat. No H1009, ≥30%), methanol (Cat. No. 322415, ≥99.8%), myoglobin (Cat. No M0630, ≥95%), potassium bromide (Cat. No 243418, ≥99%), sulphuric acid (Cat. No 339741, ≥99%), 2,4,6-tri(2-pyridyl)-1,3,5-triazine (Cat. No 93285, ≥99%), and trolox (Cat. No 238813, ≥97%).

### 3.2. Total Extract Preparation from Fragaria Fruits

For preparation of the total extract of *Fragaria* fruits, the fresh material was homogenized in a Grindomix GM 200 grinder (Retsch GmbH, Haan, Germany) and 100 g was extracted twice with stirring in a glass flask (0.5 L) with methanol (100 mL) using an ultrasonic bath Sapphire 2.8 (Sapphire Ltd., Moscow, Russia) for 30 min and at 50 °C (ultrasound power 100 W and frequency 35 kHz). The extracts were filtered through cellulose, concentrated in vacuo until dryness, and stored at 4 °C before use for chemical analysis and biological activity study. The yields of total extracts of *Fragaria* fruits were 10.63 g (*F. viridis* unripe fruits), 11.02 g (*F. viridis* intermediate ripe fruits), 12.43 g (*F. viridis* fully ripe fruits), 15.63 g (*F. vesca* fully ripe fruits), and 17.33 g (*F. ananassa* fully ripe fruits).

### 3.3. High-Performance Liquid Chromatography with Photodiode Array Detection and Electrospray Ionization Triple Quadrupole Mass Spectrometric Detection (HPLC-PDA-ESI-tQ-MS): Metabolite Profiling

Metabolite profiling of *F. viridis* extracts was realized using high-performance liquid chromatography with photodiode array detection and electrospray ionization triple quadrupole mass spectrometric detection (HPLC-PDA-ESI-tQ-MS) performed on a liquid chromatograph LC-20 Prominence coupled photodiode array detector SPD-M30A (wavelength range 200–600 nm), triple-quadrupole mass spectrometer LCMS 8050 (all Shimadzu, Columbia, MD, USA) and C18 column (GLC Mastro; 150 × 2.1 mm, Ø 3 μm; Shimadzu, Kyoto, Japan) at the column temperature 30 °C. Gradient elution was implemented with two eluents A (0.5% HCOOH in water) and B (0.5% HCOOH in MeCN) and the following gradient program: 0–5 min 5–7% B, 5–7 min 7–8% B, 7–10 min 8–19% B, 10–14 min 19–29% B, 14–20 min 29–52% B, 20–25 min 52–73% B, 25–35 min 73–90% B, and 35–45 min 90–5% B. The values of injection volume and elution flow were 1 μL and 100 μL/min, respectively. The UV-Vis spectra were obtained in the spectral range of 200–600 nm. MS detection was performed in negative ESI mode using the parameters as follows: temperature levels of ESI interface, desolvation line, and heat block were 300 °C, 250 °C, and 400 °C, respectively. The flow levels of nebulizing gas (N_2_), heating gas (air), and collision-induced dissociation gas (Ar) were 3 L/min, 10 L/min, and 0.3 mL/min, respectively. The MS spectra were recorded in the negative mode (−3–−5 kV source voltage) by scanning in the range of *m*/*z* 50–2000 at the collision energy of 5–40 eV. The system was managed under LabSolution’s workstation software with the inner LC-MS library. The identification of compounds was done by the analysis of their retention time, ultraviolet, and mass-spectrometric data, comparing the same parameters with the reference samples and/or literature data. Before analysis, the sample of *F. viridis* fruits dry extract (10 mg) was dissolved in 50% MeCN (25 mL), filtered (0.22-μm PTFE syringe filter), and injected (1 μL) into the HPLC-DAD-ESI-tQ-MS system for analysis.

### 3.4. High-Performance Liquid Chromatography with Diode Array Detection (HPLC-DAD): Carbohydrate Analysis

The composition of free carbohydrates was analyzed by high-performance liquid chromatography with diode array detection (HPLC-DAD) using the procedure described previously [69]. To prepare the sample, dry extracts of *F. viridis* fruits (5 mg) were dissolved in 20 mL of deionized water and passed sequentially through a series of two cartridges Dowex^®^ 50WX8 (H^+^-form; 10 mL) and Dowex^®^ 1 × 8 (Cl^−^-form; 10 mL) eluted with water (20 mL). The final eluates were reduced in vacuo (20 mL) and filtered using 0.22-μm PTFE syringe filter before injection into the HPLC-DAD system for analysis.

### 3.5. HPLC-ESI-tQ-MS: Metabolite Quantification

To quantify compounds **1**–**95** in *F. viridis* fruits, we used HPLC-MS data (MS peak area) obtained in early conditions (Section 3.3). The reference standards (48 compounds; Table S2) were accurately weighed (10 mg) and individually dissolved in DMSO-50% methanol mixture (1:10) in a volumetric flask (10 mL). The stock solutions were used to build external standard calibration curves generated using six data points, 100, 50, 25, 10, 5, and 1 µg/mL followed by plotting the MS peak area vs. the concentration levels. The validation criteria (correlation coefficients, *r*^2^; standard deviation, *S*_YX_; limits of detection, LOD; limits of quantification, LOQ; and linear ranges) were calculated using the previous recommendations [70] (Table S2). All analyses were carried out in triplicate, and the data were expressed as mean value ± standard deviation (S.D.). The sample solution was prepared from homogenized *F. viridis* fruits (50 mg) and 5 mL of methanol in an Eppendorf tube. The mixture was sonicated for 30 min at 50 °C (ultrasound power 100 W, frequency 35 kHz), centrifuged (6000× *g*), filtered (using 0.22-μm PTFE syringe filter), and transferred to the volumetric flask (10 mL), and the final volume was reduced to 10 mL by 50% MeOH before HPLC-ESI-tQ-MS analysis. Genkwanin was used as the internal standard (final concentration 25 μg/mL in acetonitrile).

### 3.6. Antioxidant Activity: In Vitro Assays

Radical scavenging activity of *Fragaria* extracts against the 2,2-diphenyl-1-picrylhydrazyl radical (DPPH) and the 2,2′-azino-bis(3-ethylbenzothiazoline-6-sulfonic acid) cation radical (ABTS) was studied using microplate spectrophotometric decoloration assays as described previously [71,72]. The value of the ferric reducing antioxidant power (FRAP) was measured by spectrophotometric assay based on the reduction of the Fe^3+^-2,4,6-tri(2-pyridyl)-1,3,5-triazine complex to the ferrous form at low pH [73]. To determine the level of oxygen radical absorbance capacity (ORAC), we used an assay based on peroxyl radical generation by thermal decomposition of 2,2′-azobis(2-amidino-propane) dihydrochloride followed by fluorimetric detection [74]. All assays used trolox as a reference standard (methanolic solution 0.5–100 μg/mL), and the calibration curve was created by plotting the trolox concentration (μg/mL) vs. the absorbance (or fluorescence). The values of antioxidant parameters were expressed as μmol trolox-equivalents/g of dry weight. All the analyses were carried out five times and the data were expressed as mean value ± standard deviation (SD).

### 3.7. DPPH Radical Scavenging Assisted HPLC-PDA-ESI-tQ-MS Assay

High-performance liquid chromatography with photodiode array detection and electrospray ionization triple quadrupole mass spectrometric detection (HPLC-PDA-ESI-tQ-MS) assisted with spectrophotometric DPPH radical scavenging assay was realized in the chromatographic conditions described in Section 3.3 with enlarged injection volume at 30 μL. The eluates (50 µL) were collected every 30 s using an automated fraction collector (Econova, Novosibirsk, Russia) in 96-well microplates, then dried under a N_2_-stream, and redissolved in 50 µL of 50% methanol. An aliquot (25 µL) of the methanolic solution was mixed with DPPH solution (50 µg/mL in methanol) and absorbance was measured at 520 nm fifteen minutes later by a Bio-Rad microplate reader Model 3550 UV (Bio-Rad Labs, Richmond, CA, USA). The most active antioxidants gave strong decoloration of the DPPH solution, and corresponding eluates were separated in known HPLC-PDA-ESI-tQ-MS conditions again in order to confirm the presence of separate compounds.

### 3.8. Serum Total Antioxidant Capacity

Twenty-eight men, aged 20–25 years, were recruited. All were free from hypertension, cardiovascular disorders, and alcohol abuse; none smoked or took any other drug and oral medication. We had the guarantee that all subjects had a similar diet and lifestyle because they were recruited from the same community with a refectory service. All subjects gave their informed consent for inclusion before they participated in the study. The study was conducted in accordance with the Declaration of Helsinki, and the protocol was approved by the Ethics Committee of Institute of General and Experimental Biology (protocol No. LM-0324, 27 January 2012). The volunteers were divided on six experimental groups: group 1—*F. viridis* fruits, 100 g/day (*n* = 5); group 2—*F. viridis* fruits, 250 g/day (*n* = 5); group 3—*F. viridis* fruits, 400 g/day (n = 5); group 4—*F. vesca* fruits, 250 g/day (*n* = 4); group 5—*F. ananassa* fruits, 250 g/day (*n* = 6); and group 6—fructose, 10 g/day (*n* = 3). Then, they took *Fragaria* fruits or fructose for 1 week (3 times a day in equal portions). Before and after the test, blood was drawn from the antecubital vein into a heparinized syringe, and immediately after blood drawing, serum was prepared by centrifugation (6000× *g*) and the serum total antioxidant capacity was estimated. Phosphate buffer (10 mM, pH 7.2; 100 μL), myoglobin solution (5 μM; 50 μL), ABTS solution (3 mM; 20 μL), and serum sample (20 μL) were mixed in 96-well microplate and incubated 3 min at 25 °C. Then H_2_O_2_ solution (250 μM; 20 μL) was added and immediately measured at 600 nm for 5 min at 25 °C. A lag time (in sec) was estimated as the suppression period of ABTS oxidation (or absorbance increasing). The reference compound (trolox; 1, 2.5, 5, and 10 μM) was analyzed using the same protocol, and the calibration curve was created by plotting the lag time (in s) vs. the absorbance at 600 nm. The value of the serum total antioxidant capacity was expressed as μmol trolox-equivalents/L. All the analyses were carried out in triplicate, and the data were expressed as mean value ± standard deviation (SD).

### 3.9. F. viridis Fruit Storage Experiment

Seven and three portions of the fresh *F. viridis* fruits (200 g) were placed into individual polystyrene bags (300 mL) and incubated at 4 °C (7 days) or 20 °C (3 days), respectively, in a ventilated MK 53 thermostat (BINDER GmbH, Tuttlingen, Germany). Five portions (20 g each) of fresh *F. viridis* fruits were taken out of storage for analysis every 24 h, extracted as described previously (Section 3.5), and analyzed using HPLC-ESI-tQ-MS quantitative procedure (Section 3.5) or used without pre-extraction for the total antioxidant potential determination by coulometric assay (Section 3.10).

### 3.10. Total Antioxidant Potential of Fresh F. viridis Fruits: Coulometric Assay

The total antioxidant potential of fresh *F. viridis* fruits was found using a sightly modified bromine radical scavenging assay based on the coulometric titration method with electrogenerated bromine radicals [17,75]. Potentiostat Expert-006 (Econics Expert Ltd., Moscow, Russia) with a four-electrode two-compartment electrochemical cell was used for measurements. The working electrode was a bare platinum foil (surface area 1 cm^2^), and the auxiliary electrode was a platinum wire isolated from the anodic cell with a semipermeable diaphragm. To detect the titration end-point (Δ*E* = 200 mV), a pair of polarized platinum electrodes was used and the electrochemical generation was carried out from the supporting electrolyte (0.25 M KBr in 0.1 M H_2_SO_4_) at a current density 5 mA∙cm^−2^, providing 100% current yield. To start the measurement, the portion fruit of *F. viridis* (50 g) with various storage periods was homogenized and 10 mg of homogenate was inserted into the coulometric cell (50 mL) containing 20.0 mL of supporting electrolyte. The time of titration was used for the total antioxidant potential calculation expressed in units of the quantity of electricity (Coulombs (C)) spent for titration of the full probe of homogenized fruits. The trolox solutions were used as a reference compound (500, 250, 100, 50, and 10 μg/mL in methanol) titrated coulometrically, and a calibration curve was plotted in coordinates “concentration (μg/mL)—the quantity of electricity (C)”. Finally, the value of the total antioxidant potential was calculated as mg trolox-equivalents per g of fresh fruits. Values are expressed as mean obtained from ten independent experiments.

### 3.11. Statistical Analysis

Statistical analyses were performed using a one-way analysis of variance (ANOVA), and the significance of the mean difference was determined by Duncan’s multiple range test. Differences at *p* < 0.05 were considered statistically significant. The results are presented as mean values ± S.D. (standard deviations) of some (3–10) replicates.

## 4. Conclusions

The current study reported the metabolic profile of fruits of *Fragaria viridis* in various stages of ripening using the HPLC-DAD-ESI-tQ-MS technique not applied previously to this strawberry species. About a hundred compounds were characterized, and this is many more than the previously reported amount of *F. viridis* metabolites [14,15]. The largest number of components were phenolics, particularly ellagitannins and flavonol glycosides, forming the basis of *F. viridis* metabolome in all stages of ripening. In addition, derivatives of gallic acid, ellagic acid, hydroxycinnamates, coumarins, procyanidins, catechins, and anthocyanins were also found. Non-phenolic compounds, such as carbohydrates and organic acids, were quantitatively predominant, opposite triterpenes, with trace levels found. The concentrations of all compounds were affected by the ripening process with increased (anthocyanins and non-phenolics) or decreased (the majority of phenolic compounds) values to a fully ripe stage. This indicates that the ripening of *F. viridis* fruits is a complex process impacting the quantitative profile of metabolites. The high content of ascorbic acid and selected phenolics in *F. viridis* fruits were the source of strong antioxidant properties of fruit extracts, in particular free radical scavenging capacity, ferric reducing antioxidant power, and oxygen radical absorbance capacity studied in in vitro models. The same is true for human experiments, which demonstrated that the serum total antioxidant capacity increased significantly after a week’s consumption of *F. viridis* fruits. Changes in antioxidant content and total antioxidant potential of fresh *F. viridis* fruits was found during storage at 4 °C and 20 °C, with the safest condition at 4 °C storage used within a week. The information received in our study highlighted the potential of *F. viridis* fruits as a source of antioxidant metabolites that need more scientific attention and wider implementation in the human diet.

## Figures and Tables

**Table 1:** Chromatographic (*t*_R_) and mass-spectrometric data of compounds **1**–**95** found in *F. viridis* fruits.

No.	*t*_R_, min	[M-H]^− I^, [M-2H]^− II^, [M-2H]^2− III^, *m*/*z*	MS/MS, *m*/*z*	Group ^a^	Compound [ref.] ^b^	Presence in Ripening Stages ^c^
**1**	2.51	341 ^I^		CR	Hexosyl-hexose ^L^ [16]	+/+/+
**2**	2.94	179 ^I^		CR	Hexose ^L^ [16]	+/+/+
**3**	3.08	191 ^I^		OA	Citric acid ^S^	+/+/+
**4**	3.33	133 ^I^		OA	Malic acid ^S^	+/+/+
**5**	3.41	149 ^I^		OA	Tartaric acid ^S^	+/+/+
**6**	3.50	115 ^I^		OA	Fumaric acid ^S^	+/+/+
**7**	3.82	175 ^I^		OA	Ascorbic acid ^S^	+/+/+
**8**	4.22	89 ^I^		OA	Oxalic acid ^S^	+/+/+
**9**	5.03	331 ^I^	169, 125	GA	1-*O*-Galloyl glucose ^S^ [17]	+/+/+
**10**	5.98	169 ^I^		GA	Gallic acid ^S^ [17]	+/+/+
**11**	6.71	783 ^I^; 391 ^III^	633, 481, 301	ET	Pedunculagin ^S^ [18]	+/+/+
**12**	7.03	161 ^I^		CO	Umbelliferone ^S^ [19]	+/+/+
**13**	7.43	353 ^I^	191, 179, 173, 135	HC	4-*O*-Caffeoylquinic acid ^S^ [20]	+/+/+
**14**	7.50	633 ^I^	481, 331, 301	ET	Strictinin ^S^ [18]	+/+/+
**15**	7.58	353 ^I^	191, 165	HC	5-*O*-Caffeoylquinic acid ^S^ [20]	+/+/+ [15]
**16**	7.44	633 ^I^	481, 331, 301	ET	Strictinin isomer ^L^ [18]	+/+/+
**17**	7.83	353 ^I^	191, 179, 135	HC	3-*O*-Caffeoylquinic acid ^S^ [20]	+/+/+
**18**	8.01	783 ^I^; 391 ^III^	633, 481, 301	ET	Pedunculagin isomer ^L^ [18]	+/+/+
**19**	8.85	577 ^I^	289	PC	Procyanidin B2 (catechin dimer) ^S^ [17]	+/+/+
**20**	9.15	609 ^II^	447, 285	CY	Cyanidin 3-*O*-sophoroside ^S^ [21]	−/+/+
**21**	9.48	577 ^I^	289	PC	Procyanidin B4 (catechin-epicatechin dimer) ^S^ [17]	+/+/+
**22**	9.67	593 ^II^	431, 269	CY	Pelargonidin di-*O*-hexoside ^L^ [21]	−/+/+
**23**	10.14	289 ^I^		CT	Catechin ^S^ [21]	+/+/+ [14]
**24**	10.49	325 ^I^	163, 119	HC	*p*-Coumaric acid *O*-hexoside ^L^ [22]	+/+/+
**25**	10.58	325 ^I^	163, 119	HC	*p*-Coumaric acid 4-*O*-glucoside ^S^ [22]	+/+/+
**26**	10.79	593 ^II^	447, 285	CY	Cyanidin 3-*O*-rutinoside ^S^ [21]	−/+/+
**27**	11.02	447 ^II^	285	CY	Cyanidin 3-*O*-glucoside ^S^ [21]	−/+/+ [14]
**28**	11.14	577 ^II^	431, 269	CY	Pelargonidin 3-*O*-rutinoside ^S^ [21]	−/+/+
**29**	11.52	431 ^II^	269	CY	Pelargonidin 3-*O*-glucoside ^S^ [21]	+/+/+ [14]
**30**	12.01	933 ^I^; 466 ^III^	301	ET	Castalagin ^S^ [18,23]	+/+/+
**31**	12.11	865 ^I^	577, 289	PC	Procyanidin C2 (catechin trimer) ^S^ [17]	+/+/+
**32**	12.63	593 ^II^	447, 285	CY	Cyanidin *O*-*p*-coumaroyl-*O*-hexoside ^L^ [21]	−/+/+
**33**	12.78	935 ^I^; 467 ^III^	633, 463, 301	ET	Casuarictin isomer [18]	+/+/+
**34**	12.88	933 ^I^; 466 ^III^	301	ET	Castalagin isomer ^L^ [18,23]	+/+/+
**35**	13.09	1103 ^I^; 551 ^III^	951, 933, 783, 633, 481, 301	ET	Sanguiin H2 ^S^ [18,24]	+/+/+
**36**	13.41	865 ^I^	577, 289	PC	Procyanidin trimer (catechin/epicatechin trimer) ^L^ [17]	+/+/+
**37**	13.70	577 ^II^	431, 269	CY	Pelargonidin *O*-*p*-coumaroyl-*O*-hexoside ^L^ [21]	−/+/+
**38**	13.98	489 ^II^	447, 285	CY	Cyanidin *O*-acetyl-*O*-hexoside ^L^ [21]	−/+/+
**39**	14.03	625 ^I^	463, 301	FG	Quercetin 3-*O*-sophoroside ^S^ [25]	+/+/+
**40**	14.11	935 ^I^; 467 ^III^	633, 463, 301	ET	Casuarictin isomer [18]	+/+/+
**41**	14.51	433 ^I^	301	ET	Ellagic acid *O*-pentoside ^L^ [26,27]	+/+/+
**42**	14.88	1567 ^I^; 783 ^III^	933, 633, 301	ET	Sanguiin H10 ^S^ [18,24]	+/+/+
**43**	15.01	447 ^I^	301	ET	Ellagic acid *O*-desoxyhexoside ^L^ [26,27]	+/+/+
**44**	15.11	1103 ^I^; 551 ^III^	933, 783, 633, 481, 301	ET	Sanguiin H2 isomer ^L^ [18,24]	+/+/+
**45**	15.34	609 ^I^	463, 301	FG	Quercetin 3-*O*-rutinoside ^S^ [25]	+/+/+ [15]
**46**	15.53	1869 ^I^; 934 ^III^	1567, 1265, 935, 783, 633, 481, 301	ET	Sanguiin H6 isomer ^L^ [18,24]	+/+/+
**47**	15.72	1401 ^III^	1235, 933, 783, 633, 301	ET	Lambertianin C ^S^ [18,24]	+/+/+
**48**	15.81	473 ^II^	431, 269	CY	Pelargonidin *O*-acetyl-*O*-hexoside ^L^ [21]	−/+/+
**49**	15.94	463 ^I^	301	FG	Quercetin 3-*O*-glucoside ^S^ [25]	+/+/+ [14]
**50**	16.02	477 ^I^	301	FG	Quercetin 3-*O*-glucuronide ^S^ [25]	+/+/+
**51**	16.33	1103 ^I^; 551 ^III^	801, 783, 499, 481, 319, 301	ET	Agrimonic acid A ^S^ [28]	+/+/+
**52**	16.50	1869 ^I^; 934 ^III^	1701, 1567, 1265, 1085, 935, 783, 633, 481, 301	ET	Sanguiin H6 ^S^ [18,24]	+/+/+
**53**	16.71	301 ^I^	229	ET	Ellagic acid ^S^ [18]	+/+/+ [14]
**54**	16.81	1103 ^I^; 551 ^III^	801, 783, 499, 481, 319, 301	ET	Agrimonic acid B ^S^ [28]	+/+/+
**55**	16.95	433	301	FG	Quercetin 3-*O*-xyloside ^S^ [25]	+/+/+
**56**	17.07	433	301	FG	Quercetin 3-*O*-arabinoside ^S^ [25]	+/+/+
**57**	17.41	593 ^I^	447, 285	FG	Kaempferol 3-*O*-rutinoside ^S^ [25]	+/+/+
**58**	17.56	447 ^I^	285	FG	Kaempferol 3-*O*-glucoside ^S^ [25]	+/+/+
**59**	17.92	461 ^I^	285	FG	Kaempferol 3-*O*-glucuronide ^S^ [25]	+/+/+
**60**	18.21	609 ^I^	463, 301	FG	Quercetin 3-*O*-(6″-*O*-*p*-coumaroyl)-glucoside ^S^ [25]	+/+/+
**61**	18.29	609 ^I^	463, 301	FG	Quercetin *O*-*p*-coumaroyl-*O*-hexoside ^L^ [25]	+/+/+
**62**	18.50	1869 ^I^; 934 ^III^	1567, 1265, 1085, 935, 783, 633, 481, 301	ET	Agrimoniin ^S^ [23,29]	+/+/+
**63**	18.68	1018 ^III^	1691, 1567, 1265, 1209, 935, 783, 633, 481, 301	ET	Fragariin A ^L^ [23,29]	+/+/+
**64**	19.06	549 ^I^	463, 301	FG	Quercetin *O*-malonyl-*O*-hexoside ^L^ [30,31]	+/+/+
**65**	19.42	549 ^I^	463, 301	FG	Quercetin 3-*O*-(6″-*O*-malonyl)-glucoside ^S^ [30,31]	+/+/+
**66**	19.75	593 ^I^	447, 285	FG	Kaempferol 3-*O*-(6″-*O*-*p*-coumaroyl)-glucoside ^S^ [25]	+/+/+
**67**	19.83	939 ^I^	787, 635, 483, 331, 169	GA	1,2,3,4,6-Penta-*O*-galloylglucose ^S^ [17]	+/+/+
**68**	20.39	533 ^I^	447, 285	FG	Kaempferol *O*-malonyl-*O*-hexoside ^L^ [30,31]	+/+/+
**69**	20.82	533 ^I^	447, 285	FG	Kaempferol 3-*O*-(6″-*O*-malonyl)-glucoside ^S^ [30,31]	+/+/+
**70**	21.83	505 ^I^	463, 301	FG	Quercetin 3-*O*-(2″-*O*-acetyl)-glucoside ^S^ [32]	+/+/+
**71**	22.14	505 ^I^	463, 301	FG	Quercetin 3-*O*-(6″-*O*-acetyl)-glucoside ^S^ [32]	+/+/+
**72**	22.67	489 ^I^	447, 285	FG	Kaempferol *O*-acetyl-*O*-hexoside ^L^ [30,31,32]	+/+/+
**73**	23.52	489 ^I^	447, 285	FG	Kaempferol *O*-acetyl-*O*-hexoside ^L^ [30,31,32]	+/+/+
**74**	24.21	301 ^I^		FG	Quercetin ^S^ [25]	+/+/+ [14]
**75**	24.78	811 ^I^	649, 487	TR	Tormentic acid di-*O*-hexoside ^L^ [16]	+/+/+
**76**	25.34	285 ^I^		FG	Kaempferol ^S^ [25]	+/+/+
**77**	25.54	795 ^I^	633, 471	TR	Pomolic acid di-*O*-hexoside ^L^ [16]	+/+/+
**78**	25.87	649 ^I^	487	TR	Tormentic acid *O*-hexoside ^L^ [16]	+/+/+
**79**	26.41	547 ^I^	505, 463, 301	FG	Quercetin 3-*O*-(2″,6″-di-*O*-acetyl)-glucoside ^S^ [32]	+/+/+
**80**	27.52	591 ^I^	549, 505, 463, 301	FG	Quercetin *O*-acetyl-*O*-malonyl-*O*-hexoside ^L^ [30,31,32]	+/+/+
**81**	27.73	487 ^I^		TR	Tormentic acid ^S^ [16]	+/+/+
**82**	27.89	461 ^I^	315, 301	ET	Ellagic acid *O*-methyl ester-*O*-desoxyhexoside ^L^ [26,27]	+/+/+
**83**	28.78	633 ^I^	471	TR	Pomolic acid *O*-hexoside ^L^ [16]	+/+/+
**84**	29.14	695 ^I^	609, 463, 301	FG	Quercetin *O*-malonyl-*O*-*p*-coumaroyl-*O*-hexoside ^L^ [30,31,32]	+/+/+
**85**	29.49	695 ^I^	609, 463, 301	FG	Quercetin *O*-malonyl-*O*-*p*-coumaroyl-*O*-hexoside ^L^ [30,31,32]	+/+/+
**86**	29.57	475 ^I^	329, 301	ET	Ellagic acid di-*O*-methyl ester-*O*-desoxyhexoside ^L^ [26,27]	+/+/+
**87**	30.08	531 ^I^	489, 447, 285	FG	Kaempferol di-*O*-acetyl-*O*-hexoside ^L^ [30,31,32]	+/+/+
**88**	30.41	651 ^I^	609, 463, 301	FG	Quercetin *O*-acetyl-*O*-*p*-coumaroyl-*O*-hexoside ^L^ [30,31,32]	+/+/+
**89**	30.92	575 ^I^	533, 489, 447, 285	FG	Kaempferol *O*-acetyl-*O*-malonyl-*O*-hexoside ^L^ [30,31,32]	+/+/+
**90**	31.02	679 ^I^	593, 447, 285	FG	Kaempferol *O*-malonyl-*O*-*p*-coumaroyl-*O*-hexoside ^L^ [30,31,32]	+/+/+
**91**	31.22	471 ^I^		TR	Pomolic acid ^S^ [16]	+/+/+
**92**	31.38	679 ^I^	593, 447, 285	FG	Kaempferol *O*-malonyl-*O*-*p*-coumaroyl-*O*-hexoside ^L^ [30,31,32]	+/+/+
**93**	31.98	635 ^I^	593, 447, 285	FG	Kaempferol *O*-acetyl-*O*-*p*-coumaroyl-*O*-hexoside ^L^ [30,31,32]	+/+/+
**94**	32.86	693 ^I^	651, 609, 463, 301	FG	Quercetin di-*O*-acetyl-*O*-*p*-coumaroyl-*O*-hexoside ^L^ [30,31,32]	+/+/+
**95**	34.26	737 ^I^	695, 651, 609, 463, 301	FG	Quercetin *O*-acetyl-*O*-malonyl-*O*-*p*-coumaroyl-*O*-hexoside ^L^ [30,31,32]	+/+/+

^a^ Groups of compounds: CO—coumarins; CR—carbohydrates; CY—anthocyanins; ET—ellagitannins; FG—flavonols and flavonol glycosides; GA—gallic acid derivatives; HC—hydroxycinnamates; OA—organic acids; PC—procyanidins; and TR—triterpenes. ^b^ Compound identification was based on comparison of retention time, UV and MS spectral data with reference standard (^S^), or interpretation of UV and MS spectral data and comparison with literature data (^L^). ^c^ Compounds were detected (+) or not (−) in unripe/intermediate ripe/fully ripe *F. viridis* fruits; if compound was previously reported in *F. viridis* fruits, the reference no. is mentioned.

**Table 2:** Content of compounds in unripe, intermediate ripe, and fully ripe fruits of *F. viridis*, mg/g of fresh fruit weight ± S.D.

Compound	Stage of Ripeness		
	Unripe	Intermediate	Ripe
Carbohydrates			
Hexose (glucose+fructose)	41.10 ± 0.82	43.26 ± 0.90	45.16 ± 0.92
Hexosyl-hexose (saccharose)	0.04 ± 0.00	0.06 ± 0.00	0.11 ± 0.00
Total carbohydrates	41.14	43.32	45.27
Organic acids			
Ascorbic acid	0.62 ± 0.02	0.86 ± 0.02	1.12 ± 0.02
Citric acid	2.83 ± 0.06	3.18 ± 0.07	5.63 ± 0.11
Malic acid	0.42 ± 0.01	0.45 ± 0.01	0.59 ± 0.02
Tartaric acid	0.37 ± 0.01	0.40 ± 0.01	0.42 ± 0.01
Fumaric acid	0.01 ± 0.00	0.03 ± 0.00	0.07 ± 0.00
Oxalic acid	traces	traces	0.05 ± 0.00
Total organic acids	4.25	4.92	7.88
Gallic acid derivatives			
Gallic acid	traces	0.01 ± 0.00	0.01 ± 0.00
1-*O*-Galloyl glucose	0.05 ± 0.00	0.03 ± 0.00	0.03 ± 0.00
1,2,3,4,6-Penta-*O*-galloylglucose	traces	traces	traces
Total gallic acid derivatives	0.05	0.04	0.04
Hydroxycinnamates and coumarins			
*p*-Coumaric acid 4-*O*-glucoside	0.35 ± 0.01	0.33 ± 0.01	0.29 ± 0.00
*p*-Coumaric acid *O*-hexoside **24**	0.14 ± 0.00	0.11 ± 0.00	0.08 ± 0.00
3-*O*-Caffeoylquinic acid	0.12 ± 0.00	0.08 ± 0.00	0.04 ± 0.00
4-*O*-Caffeoylquinic acid	0.08 ± 0.00	0.07 ± 0.00	0.05 ± 0.00
5-*O*-Caffeoylquinic acid	0.28 ± 0.00	0.21 ± 0.00	0.14 ± 0.00
Umbelliferone	traces	traces	traces
Total hydroxycinnamates and coumarins	0.97	0.80	0.60
Ellagic acid derivatives and ellagitannins			
Ellagic acid	0.10 ± 0.00	0.10 ± 0.00	0.12 ± 0.00
Ellagic acid *O*-pentoside **41**	0.05 ± 0.00	0.09 ± 0.00	0.11 ± 0.00
Ellagic acid *O*-desoxyhexoside **43**	0.01 ± 0.00	0.04 ± 0.00	0.07 ± 0.00
Ellagic acid *O*-methyl ester-*O*-desoxyhexoside **82**	0.14 ± 0.00	0.16 ± 0.00	0.24 ± 0.00
Ellagic acid di-*O*-methyl ester-*O*-desoxyhexoside **86**	0.10 ± 0.00	0.18 ± 0.00	0.30 ± 0.00
Pedunculagin	0.26 ± 0.00	0.30 ± 0.01	0.32 ± 0.01
Pedunculagin isomer **18**	0.05 ± 0.00	0.07 ± 0.00	0.11 ± 0.00
Strictinin isomer **14**	0.10 ± 0.00	0.10 ± 0.00	0.12 ± 0.00
Strictinin isomer **16**	0.11 ± 0.00	0.12 ± 0.00	0.18 ± 0.00
Castalagin isomer **30**	traces	0.02 ± 0.00	0.04 ± 0.00
Castalagin isomer **34**	traces	traces	0.01 ± 0.00
Casuarictin isomer **33**	traces	traces	0.02 ± 0.00
Casuarictin isomer **40**	0.06 ± 0.00	0.08 ± 0.00	0.14 ± 0.00
Sanguiin H2	traces	0.01 ± 0.00	0.05 ± 0.00
Sanguiin H2 isomer **44**	0.09 ± 0.00	0.05 ± 0.00	0.02 ± 0.00
Sanguiin H6	0.36 ± 0.01	0.25 ± 0.00	0.22 ± 0.00
Sanguiin H6 isomer **46**	0.45 ± 0.01	0.43 ± 0.01	0.40 ± 0.01
Sanguiin H10	0.21 ± 0.00	0.15 ± 0.00	0.08 ± 0.00
Lambertianin C	1.86 ± 0.04	1.42 ± 0.03	1.20 ± 0.02
Agrimonic acid A	0.02 ± 0.00	0.05 ± 0.00	0.08 ± 0.00
Agrimonic acid B	0.01 ± 0.00	0.03 ± 0.00	0.10 ± 0.00
Agrimoniin	2.63 ± 0.05	2.03 ± 0.04	1.41 ± 0.03
Fragariin A	0.93 ± 0.02	0.69 ± 0.02	0.63 ± 0.01
Total ellagic acid derivatives and ellagitannins	7.54	6.37	5.97
Catechins and procyanidins			
Catechin	0.11 ± 0.00	0.05 ± 0.00	0.05 ± 0.00
Procyanidin B2	0.09 ± 0.00	0.05 ± 0.00	0.02 ± 0.00
Procyanidin B4	0.02 ± 0.00	0.01 ± 0.00	traces
Procyanidin C2	0.05 ± 0.00	0.03 ± 0.00	0.01 ± 0.00
Procyanidin trimer **36**	0.02 ± 0.00	0.01 ± 0.00	0.01 ± 0.00
Total catechins and procyanidins	0.29	0.15	0.09
Anthocyanins			
Pelargonidin 3-*O*-glucoside	traces	0.02 ± 0.00	0.06 ± 0.00
Pelargonidin 3-*O*-rutinoside	n.d.	n.d.	0.03 ± 0.00
Pelargonidin di-*O*-hexoside **22**	n.d.	n.d.	traces
Pelargonidin *O*-acetyl-*O*-hexoside **48**	n.d.	n.d.	traces
Pelargonidin *O*-*p*-coumaroyl-*O*-hexoside **37**	n.d.	n.d.	traces
Cyanidin 3-*O*-glucoside	n.d.	0.01 ± 0.00	0.05 ± 0.00
Cyanidin 3-*O*-rutinoside	n.d.	n.d.	traces
Cyanidin 3-*O*-sophoroside	n.d.	n.d.	traces
Cyanidin *O*-acetyl-*O*-hexoside **38**	n.d.	n.d.	traces
Cyanidin *O*-*p*-coumaroyl-*O*-hexoside **32**	n.d.	n.d.	traces
Total anthocyanins	traces	0.03	0.14
Flavonols and flavonol glycosides			
Kaempferol	traces	traces	0.01 ± 0.00
Kaempferol 3-*O*-glucoside	traces	0.05 ± 0.00	0.09 ± 0.00
Kaempferol 3-*O*-glucuronide	traces	traces	0.08 ± 0.00
Kaempferol 3-*O*-rutinoside	0.28 ± 0.00	0.23 ± 0.00	0.11 ± 0.00
Kaempferol *O*-acetyl-*O*-hexoside **72**	traces	traces	traces
Kaempferol *O*-acetyl-*O*-hexoside **73**	traces	traces	traces
Kaempferol di-*O*-acetyl-*O*-hexoside **87**	traces	traces	traces
Kaempferol *O*-malonyl-*O*-hexoside **68**	0.01 ± 0.00	traces	traces
Kaempferol *O*-malonyl-*O*-hexoside **69**	0.02 ± 0.00	0.01 ± 0.00	traces
Kaempferol 3-*O*-(6″-*O*-*p*-coumaroyl)-glucoside	0.08 ± 0.00	0.04 ± 0.00	0.04 ± 0.00
Kaempferol *O*-acetyl-*O*-malonyl-*O*-hexoside **89**	0.02 ± 0.00	0.01 ± 0.00	0.01 ± 0.00
Kaempferol *O*-malonyl-*O*-*p*-coumaroyl-*O*-hexoside **90**	traces	traces	traces
Kaempferol *O*-malonyl-*O*-*p*-coumaroyl-*O*-hexoside **92**	traces	traces	traces
Kaempferol *O*-acetyl-*O*-*p*-coumaroyl-*O*-hexoside **93**	0.01 ± 0.00	0.01 ± 0.00	0.01 ± 0.00
Total kaempferol derivatives	0.42	0.35	0.35
Quercetin	traces	traces	0.02 ± 0.00
Quercetin 3-*O*-xyloside	traces	traces	0.03 ± 0.00
Quercetin 3-*O*-arabinoside	traces	traces	0.01 ± 0.00
Quercetin 3-*O*-glucoside	traces	0.04 ± 0.00	0.08 ± 0.00
Quercetin 3-*O*-glucuronide	traces	0.05 ± 0.00	0.11 ± 0.00
Quercetin 3-*O*-rutinoside	0.32 ± 0.00	0.28 ± 0.00	0.25 ± 0.00
Quercetin 3-*O*-sophoroside	0.11 ± 0.00	0.08 ± 0.00	0.03 ± 0.00
Quercetin 3-*O*-(2″-*O*-acetyl)-glucoside	0.06 ± 0.00	0.03 ± 0.00	0.01 ± 0.00
Quercetin 3-*O*-(6″-*O*-acetyl)-glucoside	0.03 ± 0.00	0.02 ± 0.00	0.02 ± 0.00
Quercetin 3-*O*-(2″,6″-di-*O*-acetyl)-glucoside	0.03 ± 0.00	0.01 ± 0.00	0.01 ± 0.00
Quercetin 3-*O*-(6″-*O*-malonyl)-glucoside	0.04 ± 0.00	0.04 ± 0.00	0.02 ± 0.00
Quercetin *O*-malonyl-*O*-hexoside **64**	0.01 ± 0.00	traces	traces
Quercetin 3-*O*-(6″-*O*-*p*-coumaroyl)-glucoside	0.11 ± 0.00	0.06 ± 0.00	0.05 ± 0.00
Quercetin *O*-*p*-coumaroyl-*O*-hexoside **61**	0.02 ± 0.00	0.02 ± 0.00	0.01 ± 0.00
Quercetin *O*-acetyl-*O*-malonyl-*O*-hexoside **80**	0.01 ± 0.00	0.01 ± 0.00	traces
Quercetin *O*-malonyl-*O*-*p*-coumaroyl-*O*-hexoside **84**	0.02 ± 0.00	0.01 ± 0.00	traces
Quercetin *O*-malonyl-*O*-*p*-coumaroyl-*O*-hexoside **85**	0.01 ± 0.00	0.01 ± 0.00	traces
Quercetin *O*-acetyl-*O*-*p*-coumaroyl-*O*-hexoside **88**	0.01 ± 0.00	traces	traces
Quercetin di-*O*-acetyl-*O*-*p*-coumaroyl-*O*-hexoside **94**	traces	traces	traces
Quercetin *O*-acetyl-*O*-malonyl-*O*-*p*-coumaroyl-*O*-hexoside **95**	0.04 ± 0.00	0.02 ± 0.00	0.01 ± 0.00
Total quercetin derivatives	0.82	0.68	0.66
Total flavonols and flavonol glycosides	1.24	1.03	1.01
Triterpenes			
Pomolic acid	traces	traces	0.01 ± 0.00
Pomolic acid *O*-hexoside **83**	traces	traces	traces
Pomolic acid di-*O*-hexoside **77**	traces	traces	traces
Tormentic acid	traces	traces	0.02 ± 0.00
Tormentic acid *O*-hexoside **78**	traces	traces	0.01 ± 0.00
Tormentic acid di-*O*-hexoside **75**	traces	traces	traces
Total triterpenes	traces	traces	0.04

**Table 3:** Antioxidant activity of *Fragaria* extracts in four assays, μM trolox-eq./g of dry weight ± S.D.

Assay ^a^	*F. viridis*			*F. vesca* (Ripe)	*F. ananassa* (Ripe)
	Unripe	Intermediate	Ripe		
DPPH	29.2 ± 0.6 ^d,e^	28.4 ± 0.5 ^c^	27.5 ± 0.5 ^c,d^	15.2 ± 0.3 ^b^	9.3 ± 0.2 ^a^
ABTS	35.1 ± 0.8 ^h,i^	35.3 ± 0.8 ^h^	36.2 ± 0.9 ^i^	19.7 ± 0.4 ^f,g^	14.7 ± 0.3 ^f^
FRAP	42.6 ± 1.0 ^l^	45.4 ± 1.0 ^l,m^	47.1 ± 1.0 ^m^	27.1 ± 0.5 ^k^	21.1 ± 0.4 ^j^
ORAC	33.6 ± 0.8 ^p,q^	32.8 ± 0.7 ^o,p^	33.0 ± 0.8 ^p^	25.1 ± 0.5 ^n,o^	18.9 ± 0.4 ^n^

^a^ DPPH—scavenging capacity against 2,2-diphenyl-1-picrylhydrazyl radical; ABTS—scavenging capacity against 2,2′-azino-bis(3-ethylbenzothiazoline-6-sulfonic acid) cation radical; FRAP—ferric reducing antioxidant power; and ORAC—oxygen radical absorbance capacity. Averages ± standard deviation (S.D.) were obtained from five different experiments. Values with different letters (a–q) indicate statistically significant differences among groups at *p* < 0.05 by one-way ANOVA.

**Table 4:** Content of selected antioxidants in ripe fruits of *F. viridis* (mg/g of fresh fruit weight ± S.D.) and its total antioxidant potential (coulometric titration assay; μmol trolox-eq./g of fresh fruit weight ± S.D.) after storage at 4 °C (1 week) and 20 °C (3 days).

Compound	T, °C	Day of Storage							
		0	1	2	3	4	5	6	7
Ascorbic acid	4	1.14 ± 0.02	1.02 ± 0.02	0.95 ± 0.02	0.89 ± 0.02	0.86 ± 0.02	0.73 ± 0.02	0.55 ± 0.02	0.51 ± 0.02
	20		0.85 ± 0.02	0.54 ± 0.01	0.36 ± 0.01	n.a.	n.a.	n.a.	n.a.
Ellagic acid	4	0.10 ± 0.00	0.10 ± 0.00	0.10 ± 0.00	0.12 ± 0.00	0.14 ± 0.00	0.15 ± 0.00	0.17 ± 0.00	0.19 ± 0.00
	20		0.10 ± 0.00	0.12 ± 0.00	0.25 ± 0.00	n.a.	n.a.	n.a.	n.a.
Pedunculagin	4	0.33 ± 0.01	0.34 ± 0.01	0.34 ± 0.01	0.35 ± 0.01	0.35 ± 0.01	0.38 ± 0.01	0.38 ± 0.01	0.40 ± 0.01
	20		0.35 ± 0.01	0.38 ± 0.01	0.49 ± 0.01	n.a.	n.a.	n.a.	n.a.
Sanguiin H6	4	0.20 ± 0.00	0.20 ± 0.00	0.20 ± 0.00	0.21 ± 0.00	0.22 ± 0.00	0.24 ± 0.00	0.25 ± 0.00	0.25 ± 0.00
	20		0.20 ± 0.00	0.21 ± 0.00	0.30 ± 0.00	n.a.	n.a.	n.a.	n.a.
Lambertianin C	4	1.26 ± 0.02	1.24 ± 0.02	1.20 ± 0.02	1.15 ± 0.02	1.11 ± 0.02	0.99 ± 0.02	0.97 ± 0.02	0.93 ± 0.02
	20		1.04 ± 0.02	0.90 ± 0.02	0.72 ± 0.02	n.a.	n.a.	n.a.	n.a.
Agrimoniin	4	1.47 ± 0.03	1.45 ± 0.03	1.40 ± 0.03	1.37 ± 0.03	1.35 ± 0.03	1.25 ± 0.02	1.22 ± 0.02	1.17 ± 0.02
	20		1.33 ± 0.03	1.08 ± 0.02	0.93 ± 0.02	n.a.	n.a.	n.a.	n.a.
Fragariin A	4	0.65 ± 0.02	0.65 ± 0.02	0.62 ± 0.02	0.60 ± 0.02	0.59 ± 0.01	0.55 ± 0.01	0.53 ± 0.02	0.51 ± 0.01
	20		0.42 ± 0.01	0.34 ± 0.01	0.22 ± 0.00	n.a.	n.a.	n.a.	n.a.
Pelargonidin 3-*O*-glucoside	4	0.07 ± 0.00	0.07 ± 0.00	0.07 ± 0.00	0.07 ± 0.00	0.06 ± 0.00	0.06 ± 0.00	0.05 ± 0.00	0.05 ± 0.00
	20		0.04 ± 0.00	0.02 ± 0.00	traces	n.a.	n.a.	n.a.	n.a.
Cyanidin 3-*O*-glucoside	4	0.05 ± 0.00	0.05 ± 0.00	0.05 ± 0.00	0.04 ± 0.00	0.04 ± 0.00	0.02 ± 0.00	0.02 ± 0.00	0.02 ± 0.00
	20		0.02 ± 0.00	traces	traces	n.a.	n.a.	n.a.	n.a.
Quercetin 3-*O*-glucoside	4	0.10 ± 0.00	0.10 ± 0.00	0.10 ± 0.00	0.10 ± 0.00	0.10 ± 0.00	0.10 ± 0.00	0.11 ± 0.00	0.11 ± 0.00
	20		0.10 ± 0.00	0.11 ± 0.00	0.12 ± 0.00	n.a.	n.a.	n.a.	n.a.
Quercetin 3-*O*-glucuronide	4	0.15 ± 0.00	0.15 ± 0.00	0.15 ± 0.00	0.16 ± 0.00	0.17 ± 0.00	0.17 ± 0.00	0.17 ± 0.00	0.17 ± 0.00
	20		0.15 ± 0.00	0.15 ± 0.00	0.17 ± 0.00	n.a.	n.a.	n.a.	n.a.
Quercetin 3-*O*-rutinoside	4	0.24 ± 0.00	0.24 ± 0.00	0.24 ± 0.00	0.24 ± 0.00	0.24 ± 0.00	0.22 ± 0.00	0.21 ± 0.00	0.20 ± 0.00
	20		0.24 ± 0.00	0.20 ± 0.00	0.18 ± 0.00	n.a.	n.a.	n.a.	n.a.
Total antioxidant potential	4	4.12 ± 0.09	4.10 ± 0.08	4.07 ± 0.08	4.02 ± 0.08	3.97 ± 0.08	3.86 ± 0.08	3.59 ± 0.07	3.27 ± 0.07
	20		2.88 ± 0.05	1.72 ± 0.04	0.52 ± 0.02	n.a.	n.a.	n.a.	n.a.

n.a.—not analyzed.

## Supplementary Materials

The following are available online at https://www.mdpi.com/1424-8247/13/9/262/s1, Figure S1: High-performance liquid chromatography with diode array detection chromatogram of free sugars in F. viridis ripe fruits, Figure S2: High-performance liquid chromatography with electrospray ionization triple quadrupole mass spectrometric detection chromatogram of F. viridis ripe fruits extract coupled with spectrophotometric DPPH radical scavenging assay, Table S1: Reference standards used for the qualitative and quantitative analysis by HPLC-DAD-ESI-tQ-MS assays, Table S2: Regression equations, correlation coefficients, standard deviation, limits of detection, limits of quantification, and linear ranges for 48 reference standards.
