# Supplementary material for: Fragaria viridis Fruit Metabolites: Variation of LC-MS Profile and Antioxidant Potential during Ripening and Storage
Source: Pharmaceuticals (Basel). 2020 Sep 22;13(9):262. doi: 10.3390/ph13090262 (PMC7559413; doi:10.3390/ph13090262)
Supplement: Supplementary file 1 [file pharmaceuticals-13-00262-s001.pdf]

pharmaceuticals

MDPI

Supplemetary materials

Fragaria viridis Fruit Metabolites: Variation of LC-MS Profile and Antioxidant Potential during Ripening and Storage

Daniil N. Olennikov [1,*], Aina G. Vasilieva[2], and Nadezhda K. Chirikova [2]

[1] Laboratory of Medical and Biological Research, Institute of General and Experimental Biology, Siberian Division, Russian Academy of Science, 6 Sakh'yanovoy Street, Ulan-Ude 670047, Russia

[2] Department of Biology, Institute of Natural Sciences, North-Eastern Federal University, 58 Belinsky Street, Yakutsk 677027, Russia; hofnung@mail.ru (N.K.C.)

* Correspondence: olennikovdn@mail.ru; Tel.: +7-9021-600-627 (D.N.O.)

Content

Figure S1. High-Performance Liquid Chromatography with Diode Array Detection chromatogram of free sugars in *F. viridis* ripe fruits.

Figure S2. High-Performance Liquid Chromatography with Electrospray Ionization Triple Quadrupole Mass Spectrometric Detection chromatogram of *F. viridis* ripe fruits extract coupled with spectrophotometric DPPH radical scavenging assay.

Table S1. Reference standards used for the qualitative and quantitative analysis by HPLC-DAD-ESI-tQ-MS assays.

Table S2. Regression equations, correlation coefficients, standard deviation, limits of detection, limits of quantification, and linear ranges for 48 reference standards.

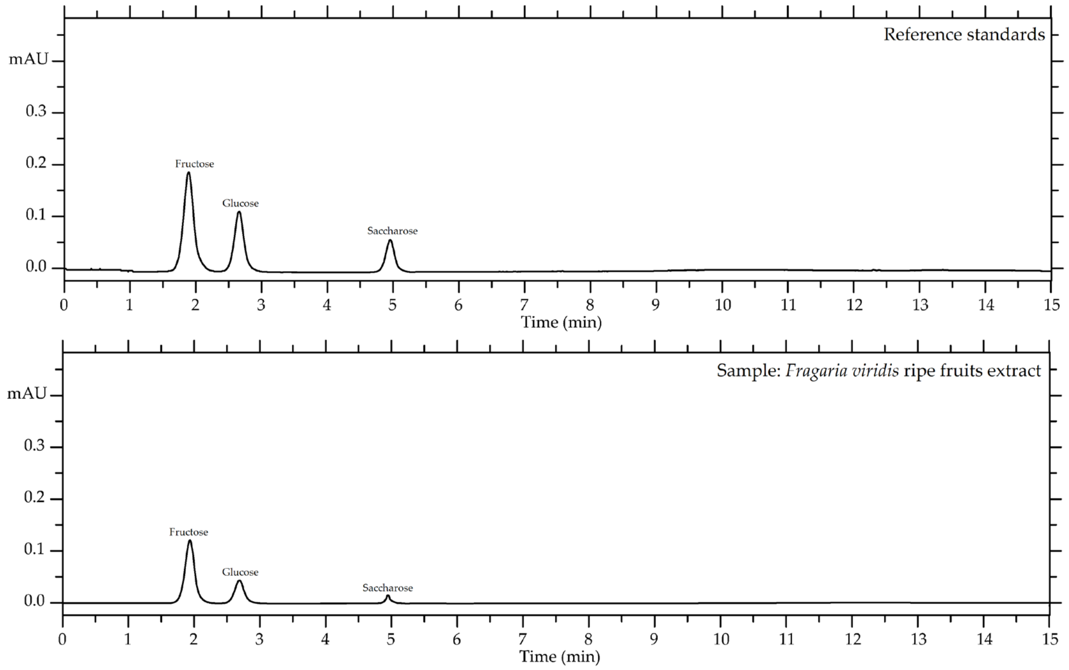

Figure S1. High-Performance Liquid Chromatography with Diode Array Detection (HPLC-DAD) chromatogram (detector wavelength 190 nm) of free sugars in *F. viridis* ripe fruits.

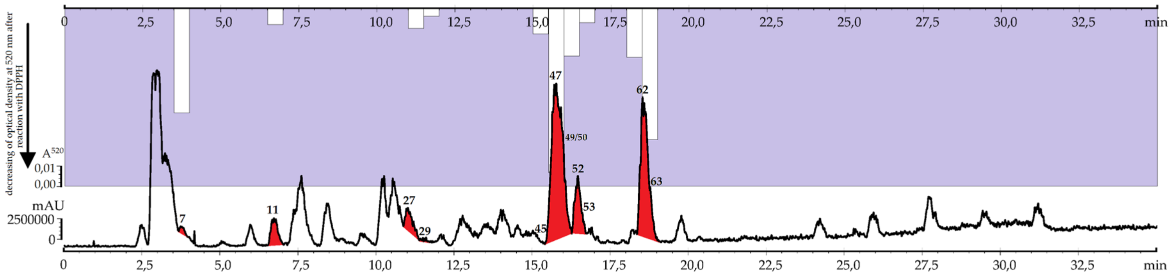

Figure S2. High-Performance Liquid Chromatography with Electrospray Ionization Triple Quadrupole Mass Spectrometric Detection (HPLC-ESI-tQ-MS) chromatogram (Total Ion Chromatogram or TIC mode, negative ionization) of *F. viridis* ripe fruits extract coupled with spectrophotometric DPPH radical scavenging assay.

Table S1. Reference standards used for the qualitative and quantitative analysis by HPLC-DAD-ESI-tQ-MS assays.

**No** [a]	**Compound**	**Standard** [a]	**Purity, (≥) %**	**Manufacturer (Cat. no)** [b]
**1**	Hexosyl-hexose	Sucrose [B]	99	Sigma (S7903)
**2**	Hexose	Fructose [A,B]	99	Sigma (47739)
**3**	Citric acid	Citric acid [A,B]	99	Sigma (251275)
**4**	Malic acid	Malic acid [A,B]	99	Sigma (W265501)
**5**	Tartaric acid	Tartaric acid [A,B]	99	Sigma (W304401)
**6**	Fumaric acid	Fumaric acid [A,B]	99	Sigma (47910)
**7**	Ascorbic acid	Ascorbic acid [A,B]	99	Sigma (255564)
**8**	Oxalic acid	Oxalic acid [A,B]	99	Sigma (658537)
**9**	1-*O*-Galloyl glucose	1-*O*-Galloyl glucose [A,B]	90	Sigma (69288)
**10**	Gallic acid	Gallic acid [A,B]	97	Sigma (G7384)
**11**	Pedunculagin	Pedunculagin [A,B]	95	Toronto (P354070)
**12**	Umbelliferone	Umbelliferone [A,B]	99	Sigma (H24003)
**13**	4-*O*-Caffeoylquinic acid	4-*O*-Caffeoylquinic acid [A,B]	98	Sigma (65969)
**14**	Strictinin	Strictinin [A,B]	98	Funakoshi (NH026102)
**15**	5-*O*-Caffeoylquinic acid	5-*O*-Caffeoylquinic acid [A,B]	95	Sigma (C3878)
**16**	Strictinin isomer	Strictinin [B]	98	Funakoshi (NH026102)
**17**	3-*O*-Caffeoylquinic acid	3-*O*-Caffeoylquinic acid [A,B]	98	Sigma (94419)
**18**	Pedunculagin isomer	Pedunculagin [B]	95	Toronto (P354070)
**19**	Procyanidin B2	Procyanidin B2 [A,B]	90	Sigma (PHL89552)
**20**	Cyanidin 3-*O*-sophoroside	Cyanidin 3-*O*-sophoroside [A,B]	85	Sigma (42739)
**21**	Procyanidin B4	Procyanidin B4 [A,B]	98	ChemFaces (CFN91171)
**22**	Pelargonidin di-*O*-hexoside	Pelargonidin 3-*O*-rutinoside [B]	98	ChemFaces (CFN92133)
**23**	Catechin	Catechin [A,B]	95	Sigma (PHL89172)
**24**	*p*-Coumaric acid *O*-hexoside	*p*-Coumaric acid 4-*O*-glucoside [B]	95	Carbosynth (MC71595)
**25**	*p*-Coumaric acid 4-*O*-glucoside	*p*-Coumaric acid 4-*O*-glucoside [A,B]	95	Carbosynth (MC71595)
**26**	Cyanidin 3-*O*-rutinoside	Cyanidin 3-*O*-rutinoside [A,B]	90	Sigma (PHL80577)
**27**	Cyanidin 3-*O*-glucoside	Cyanidin 3-*O*-glucoside [A,B]	98	Sigma (94099)
**28**	Pelargonidin 3-*O*-rutinoside	Pelargonidin 3-*O*-rutinoside [A,B]	98	ChemFaces (CFN92133)
**29**	Pelargonidin 3-*O*-glucoside	Pelargonidin 3-*O*-glucoside [A,B]	98	ChemFaces (CFN92134)
**30**	Castalagin isomer	Castalagin [B]	95	Sigma (61221)
**31**	Procyanidin C2	Procyanidin C2 [A,B]	95	TransMIT (C6201-P045)
**32**	Cyanidin *O*-*p*-coumaroyl-*O*-hexoside	Cyanidin 3-*O*-glucoside [B]	98	Sigma (94099)
**33**	Casuarictin isomer	Pedunculagin [B]	95	Toronto (P354070)
**34**	Castalagin isomer	Castalagin [B]	95	Sigma (61221)
**35**	Sanguiin H2	Sanguiin H2 [A]<br>Pedunculagin [B]	92<br>95	[68]<br>Toronto (P354070)
**36**	Procyanidin trimer	Procyanidin C2 [B]	95	TransMIT (C6201-P045)
**37**	Pelargonidin *O*-*p*-coumaroyl-*O*-hexoside	Pelargonidin 3-*O*-glucoside [B]	98	ChemFaces (CFN92134)
**38**	Cyanidin *O*-acetyl-*O*-hexoside	Cyanidin 3-*O*-glucoside [B]	98	Sigma (94099)
**39**	Quercetin 3-*O*-sophoroside	Quercetin 3-*O*-sophoroside [A,B]	98	ChemFaces (CFN90630)
**40**	Casuarictin isomer	Pedunculagin [B]	95	Toronto (P354070)
**41**	Ellagic acid *O*-pentoside	Ellagic acid [B]	98	ChemFaces (CFN98716)
**42**	Sanguiin H10	Sanguiin H10 [A]<br>Pedunculagin [B]	92<br>95	[68]<br>Toronto (P354070)
**43**	Ellagic acid *O*-desoxyhexoside	Ellagic acid [B]	98	ChemFaces (CFN98716)
**44**	Sanguiin H2 isomer	Sanguiin H2 [A]<br>Pedunculagin [B]	92<br>95	[68]<br>Toronto (P354070)
**45**	Quercetin 3-*O*-rutinoside	Rutin	95	Sigma (R2303)
**46**	Sanguiin H6 isomer	Sanguiin H6 [A]<br>Pedunculagin [B]	92<br>95	[68]<br>Toronto (P354070)

Table S1. Continuation

No [a]	Compound	Standard [a]	Purity, (≥) %	Manufacturer (Cat. no) [b]
**47**	Lambertianin C	Lambertianin C [A]<br>Pedunculagin [B]	92<br>95	[68]<br>Toronto (P354070)
**48**	Pelargonidin *O*-acetyl-*O*-hexoside	Pelargonidin 3-*O*-glucoside [B]	98	ChemFaces (CFN92134)
**49**	Quercetin 3-*O*-glucoside	Quercetin 3-*O*-glucoside [A,B]	98	Sigma (16654)
**50**	Quercetin 3-*O*-glucuronide	Quercetin 3-*O*-glucuronide [A,B]	90	Sigma (90733)
**51**	Agrimonic acid A	Agrimonic acid A [A]<br>Agrimoniin [B]	90<br>95	[28]<br>[28]
**52**	Sanguiin H6	Sanguiin H6 [A]<br>Pedunculagin [B]	92<br>95	[68]<br>Toronto (P354070)
**53**	Ellagic acid	Ellagic acid	98	ChemFaces (CFN98716)
**54**	Agrimonic acid B	Agrimonic acid B [A]<br>Agrimoniin [B]	90<br>95	[28]<br>[28]
**55**	Quercetin 3-*O*-xyloside	Quercetin 3-*O*-xyloside [A,B]	97	Sigma (83390)
**56**	Quercetin 3-*O*-arabinoside	Quercetin 3-*O*-arabinoside [A,B]	95	Sigma (75759)
**57**	Kaempferol 3-*O*-rutinoside	Kaempferol 3-*O*-rutinoside [A,B]	98	Sigma (90242)
**58**	Kaempferol 3-*O*-glucoside	Kaempferol 3-*O*-glucoside [A,B]	97	Sigma (79851)
**59**	Kaempferol 3-*O*-glucuronide	Kaempferol 3-*O*-glucuronide [A,B]	98	ChemFaces (CFN90359)
**60**	Quercetin 3-*O*-(6′′-*O*-*p*-coumaroyl)-glucoside	Helichrysoside [A,B]	98	BioCrick (BCN9477)
**61**	Quercetin *O*-*p*-coumaroyl-*O*-hexoside	Helichrysoside [B]	98	BioCrick (BCN9477)
**62**	Agrimoniin	Agrimoniin [A,B]	95	[28]
**63**	Fragariin A	Agrimoniin [B]	95	[28]
**64**	Quercetin *O*-malonyl-*O*-hexoside	Quercetin 3-*O*-(6′′-*O*-malonyl)-glucoside [B]	85	Sigma (16733)
**65**	Quercetin 3-*O*-(6′′-*O*-malonyl)-glucoside	Quercetin 3-*O*-(6′′-*O*-malonyl)-glucoside [A,B]	85	Sigma (16733)
**66**	Kaempferol 3-*O*-(6′′-*O*-*p*-coumaroyl)-glucoside	Tiliroside [A,B]	98	Sigma (79257)
**67**	1,2,3,4,6-Penta-*O*-galloylglucose	1,2,3,4,6-Penta-*O*-galloyl glucose [A,B]	96	Sigma (67548)
**68**	Kaempferol *O*-malonyl-*O*-hexoside	Kaempferol 3-*O*-(6′′-*O*-malonyl)-glucoside [B]	95	BOC (81149-02-2)
**69**	Kaempferol 3-*O*-(6′′-*O*-malonyl)-glucoside	Kaempferol 3-*O*-(6′′-*O*-malonyl)-glucoside [A,B]	95	BOC (81149-02-2)
**70**	Quercetin 3-*O*-(2′′-*O*-acetyl)-glucoside	Quercetin 3-*O*-(2′′-*O*-acetyl)-glucoside [A]<br>Quercetin 3-*O*-(6′′-*O*-acetyl)-glucoside [B]	92<br>85	[70]<br>Extrasynthese (1099)
**71**	Quercetin 3-*O*-(6′′-*O*-acetyl)-glucoside	Quercetin 3-*O*-(6′′-*O*-acetyl)-glucoside [A,B]	85	Extrasynthese (1099)
**72**	Kaempferol *O*-acetyl-*O*-hexoside	Kaempferol 3-*O*-glucoside [B]	97	Sigma (79851)
**73**	Kaempferol *O*-acetyl-*O*-hexoside	Kaempferol 3-*O*-glucoside [B]	97	Sigma (79851)
**74**	Quercetin	Quercetin [A,B]	95	Sigma (Q4951)
**75**	Tormentic acid di-*O*-hexoside	Tormentic acid [A,B]	98	ChemFaces (CFN99434)
**76**	Kaempferol	Kaempferol [A,B]	99	Sigma (96353)
**77**	Pomolic acid di-*O*-hexoside	Pomolic acid [B]	98	ChemFaces (CFN99433)
**78**	Tormentic acid *O*-hexoside	Tormentic acid [B]	98	ChemFaces (CFN99434)
**79**	Quercetin 3-*O*-(2′′,6′′-di-*O*-acetyl)-glucoside	Quercetin 3-*O*-(2′′,6′′-di-*O*-acetyl)-glucoside<br>Quercetin 3-*O*-(6′′-*O*-acetyl)-glucoside [B]	90<br>85	[70]<br>Extrasynthese (1099)

Table S1. Continuation

No [a]	Compound	Standard [a]	Purity, (≥) %	Manufacturer (Cat. no) [b]
**80**	Quercetin *O*-acetyl-*O*-malonyl-*O*-hexoside	Quercetin 3-*O*-(6′′-*O*-malonyl)-glucoside [B]	85	Sigma (16733)
**81**	Tormentic acid	Tormentic acid [A,B]	98	ChemFaces (CFN99434)
**82**	Ellagic acid *O*-methyl ester-*O*-desoxyhexoside	Ellagic acid [A,B]	98	ChemFaces (CFN98716)
**83**	Pomolic acid *O*-hexoside	Pomolic acid [A,B]	98	ChemFaces (CFN99433)
**84**	Quercetin *O*-malonyl-*O*-*p*-coumaroyl-*O*-hexoside	Helichrysoside [B]	98	BioCrick (BCN9477)
**85**	Quercetin *O*-malonyl-*O*-*p*-coumaroyl-*O*-hexoside	Helichrysoside [B]	98	BioCrick (BCN9477)
**86**	Ellagic acid di-*O*-methyl ester-*O*-desoxyhexoside	Ellagic acid [A,B]	98	ChemFaces (CFN98716)
**87**	Kaempferol di-*O*-acetyl-*O*-hexoside	Kaempferol 3-*O*-glucoside [B]	97	Sigma (79851)
**88**	Quercetin *O*-acetyl-*O*-*p*-coumaroyl-*O*-hexoside	Helichrysoside [B]	98	BioCrick (BCN9477)
**89**	Kaempferol *O*-acetyl-*O*-malonyl-*O*-hexoside	Kaempferol 3-*O*-(6′′-*O*-malonyl)-glucoside [B]	95	BOC (81149-02-2)
**90**	Kaempferol *O*-malonyl-*O*-*p*-coumaroyl-*O*-hexoside	Tiliroside [B]	98	Sigma (79257)
**91**	Pomolic acid	Pomolic acid [A,B]	98	ChemFaces (CFN99433)
**92**	Kaempferol *O*-malonyl-*O*-*p*-coumaroyl-*O*-hexoside	Tiliroside [B]	98	Sigma (79257)
**93**	Kaempferol *O*-acetyl-*O*-*p*-coumaroyl-*O*-hexoside	Tiliroside [B]	98	Sigma (79257)
**94**	Quercetin di-*O*-acetyl-*O*-*p*-coumaroyl-*O*-hexoside	Helichrysoside [B]	98	BioCrick (BCN9477)
**95**	Quercetin *O*-acetyl-*O*-malonyl-*O*-*p*-coumaroyl-*O*-hexoside	Helichrysoside [B]	98	BioCrick (BCN9477)

[a] Standards were used in qualitative ([A]) or/and quantitative analysis ([B]). [b] Manufacturers list: BioCrick—BioCrick (Chengdu, Sichuan, PRC); BOC—BOC Sciences (Shirley, NY, USA); Carbosynth—Carbosynth Ltd. (Compton, UK); ChemFaces—ChemFaces (Wuhan, Hubei, PRC); Extrasynthese—Extrasynthese (Lyon, France); Funakoshi—Funakoshi Co. Ltd. (Tokyo, Japan); Sigma—Sigma-Aldrich (St. Louis, MO, USA); Toronto—Toronto Research Chemicals (North York, ON, Canada); TransMIT—TransMIT GmbH (Gießen, Germany).

Table S2. Regression equations, correlation coefficients (r^2), standard deviation (S_{YX}), limits of detection (LOD), limits of quantification (LOQ) and linear ranges for 48 reference standards.

Compound	Ionization [a]	CE [b] (eV)	Regression equation [c]		r^2	S_{YX}	LOD/LOQ (µg/mL)	Linear range (µg/mL)
			a	$b \cdot 10^6$				
1,2,3,4,6-Penta-*O*-galloyl glucose	N	-25	2.4561	-0.0171	0.9979	$12.33 \cdot 10^{-2}$	0.17/0.50	0.6–100.0
1-*O*-Galloyl glucose	N	-20	1.3586	-0.0663	0.9987	$9.69 \cdot 10^{-2}$	0.24/0.71	0.8–100.0
3-*O*-Caffeoylquinic acid	N	-15	0.9320	-0.0523	0.9991	$4.14 \cdot 10^{-2}$	0.15/0.44	0.5–100.0
4-*O*-Caffeoylquinic acid	N	-15	0.9217	-0.0437	0.9982	$3.94 \cdot 10^{-2}$	0.14/0.43	0.5–100.0
5-*O*-Caffeoylquinic acid	N	-15	0.9406	-0.0497	0.9973	$5.18 \cdot 10^{-2}$	0.18/0.55	0.6–100.0
Agrimoniin	N	-35	0.8214	-0.2716	0.9893	$5.37 \cdot 10^{-2}$	0.22/0.65	0.7–100.0
Ascorbic acid	N	-10	0.9214	-0.0373	0.9997	$2.10 \cdot 10^{-2}$	0.07/0.22	0.3–100.0
Castalagin	N	-35	0.9361	-0.4518	0.9870	$9.35 \cdot 10^{-2}$	0.32/1.00	1.0–100.0
Catechin	N	-35	0.9562	-0.0521	0.9971	$7.79 \cdot 10^{-2}$	0.27/0.82	0.9–100.0
Citric acid	N	-10	0.9518	-0.0267	0.9990	$1.03 \cdot 10^{-2}$	0.03/0.10	0.1–100.0
Cyanidin 3-*O*-glucoside	N	-20	1.4267	-0.5637	0.9907	$12.72 \cdot 10^{-2}$	0.29/0.89	0.9–100.0
Cyanidin 3-*O*-rutinoside	N	-20	1.6341	-0.4283	0.9900	$15.02 \cdot 10^{-2}$	0.30/0.92	1.0–100.0
Cyanidin 3-*O*-sophoroside	N	-20	1.5963	-0.3518	0.9910	$14.63 \cdot 10^{-2}$	0.30/0.92	1.0–100.0
Ellagic acid	N	-30	0.9114	-0.6312	0.9887	$6.37 \cdot 10^{-2}$	0.23/0.70	0.7–100.0
Fructose	N	-10	1.5632	-0.0376	0.9983	$5.14 \cdot 10^{-2}$	0.11/0.33	0.4–100.0
Fumaric acid	N	-10	0.8615	-0.0364	0.9982	$2.03 \cdot 10^{-2}$	0.03/0.07	0.1–100.0
Gallic acid	N	-20	2.6538	-0.1376	0.9990	$1.17 \cdot 10^{-2}$	0.01/0.04	0.1–100.0
Helichrysoside	N	-30	2.0319	-0.3615	0.9811	$10.09 \cdot 10^{-2}$	0.17/0.52	0.6–100.0
Kaempferol 3-*O*-(6′′-*O*-malonyl)-glucoside	N	-30	2.3618	-0.5214	0.9873	$11.35 \cdot 10^{-2}$	0.16/0.48	0.5–100.0
Kaempferol 3-*O*-glucoside	N	-20	2.0859	-0.9171	0.9980	$6.18 \cdot 10^{-2}$	0.03/0.09	0.1–100.0
Kaempferol 3-*O*-glucuronide	N	-30	2.2126	-0.5160	0.9987	$8.11 \cdot 10^{-2}$	0.12/0.37	0.4–100.0
Kaempferol 3-*O*-rutinoside	N	-30	1.9634	-0.4511	0.9952	$9.18 \cdot 10^{-2}$	0.15/0.46	0.5–100.0
Kaempferol	N	-20	1.2416	-0.3615	0.9901	$3.02 \cdot 10^{-2}$	0.08/0.24	0.3–100.0
Malic acid	N	-10	0.9911	-0.0379	0.9988	$2.05 \cdot 10^{-2}$	0.07/0.21	0.3–100.0
Oxalic acid	N	-10	0.9804	-0.0210	0.9970	$2.01 \cdot 10^{-2}$	0.06/0.21	0.3–100.0
p-Coumaric acid 4-*O*-glucoside	N	-20	1.4238	-0.0891	0.9901	$7.33 \cdot 10^{-2}$	0.17/0.52	0.6–100.0
Pedunculagin	N	-35	0.6370	-0.4521	0.9872	$6.11 \cdot 10^{-2}$	0.32/0.96	1.0–100.0
Pelargonidin 3-*O*-glucoside	N	-20	0.9634	-0.8634	0.9832	$10.37 \cdot 10^{-2}$	0.36/1.07	1.1–100.0
Pelargonidin 3-*O*-rutinoside	N	-20	0.8237	-0.7310	0.9801	$14.73 \cdot 10^{-2}$	0.59/1.79	2.0–100.0
Pomolic acid	N	-30	1.4784	-0.8634	0.9763	$19.39 \cdot 10^{-2}$	0.43/1.31	1.5–100.0
Procyanidin B2	N	-25	1.3620	-0.0820	0.9961	$9.91 \cdot 10^{-2}$	0.21/0.72	0.8–100.0
Procyanidin B4	N	-25	1.0634	-0.0933	0.9902	$10.01 \cdot 10^{-2}$	0.31/0.94	1.0–100.0
Procyanidin C2	N	-25	1.4632	-0.0524	0.9953	$8.12 \cdot 10^{-2}$	0.18/0.56	0.6–100.0
Quercetin 3-*O*-(6′′-*O*-acetyl)-glucoside	N	-20	1.1103	-0.9217	0.9901	$14.33 \cdot 10^{-2}$	0.42/1.29	1.5–100.0
Quercetin 3-*O*-(6′′-*O*-malonyl)-glucoside	N	-20	1.2703	-0.7911	0.9814	$15.26 \cdot 10^{-2}$	0.40/1.20	1.5–100.0
Quercetin 3-*O*-arabinoside	N	-20	1.4412	-0.6211	0.9930	$11.25 \cdot 10^{-2}$	0.26/0.78	0.8–100.0
Quercetin 3-*O*-glucoside	N	-20	1.8267	-0.4160	0.9990	$11.73 \cdot 10^{-2}$	0.21/0.67	0.7–100.0
Quercetin 3-*O*-glucuronide	N	-20	1.6705	-0.4374	0.9988	$12.79 \cdot 10^{-2}$	0.25/0.77	0.8–100.0
Quercetin 3-*O*-sophoroside	N	-25	1.4001	-0.8214	0.9884	$12.08 \cdot 10^{-2}$	0.29/0.86	1.0–100.0
Quercetin 3-*O*-xyloside	N	-20	1.5364	-0.3614	0.9927	$10.07 \cdot 10^{-2}$	0.22/0.66	0.7–100.0
Quercetin	N	-15	1.1105	-0.3211	0.9937	$4.18 \cdot 10^{-2}$	0.12/0.38	0.4–100.0
Rutin	N	-25	1.2716	-0.7389	0.9897	$9.14 \cdot 10^{-2}$	0.23/0.72	0.8–100.0
Strictinin	N	-35	0.9634	-0.3518	0.9804	$7.34 \cdot 10^{-2}$	0.25/0.76	0.8–100.0
Sucrose	N	-10	1.6278	-0.0428	0.9990	$7.11 \cdot 10^{-2}$	0.14/0.44	0.5–100.0

Table S2. Continuation

**Compound**	**Ionization** [a]	**CE** [b] **(eV)**	**Regression equation** [c]		r^2	S_{YX}	**LOD/ LOQ (µg/mL)**	**Linear range (µg/mL)**
			a	***b*·10⁶**				
Tartaric acid	N	-10	1.5330	-0.0863	0.9985	$4.15 \cdot 10^{-2}$	0.09/0.27	0.3–100.0
Tiliroside	N	-30	2.3312	-0.4563	0.9803	$14.92 \cdot 10^{-2}$	0.21/0.64	0.7–100.0
Tormentic acid	N	-30	1.2820	-0.9634	0.9697	$11.64 \cdot 10^{-2}$	0.30/0.91	1.0–100.0
Umbelliferone	N	-15	0.5697	-0.2634	0.9900	$4.33 \cdot 10^{-2}$	0.25/0.76	0.8–100.0

[a] Ionization mode : N—negative. [b] CE—collision energy. [c] Regression equation: $y = a \cdot x + b$.

© 2020 by the authors. Submitted for possible open access publication under the terms and conditions of the Creative Commons Attribution (CC BY) license (http://creativecommons.org/licenses/by/4.0/).
